# Hepatic Stress Response in HCV Infection Promotes STAT3-Mediated Inhibition of HNF4A-*miR-122* Feedback Loop in Liver Fibrosis and Cancer Progression

**DOI:** 10.3390/cancers11101407

**Published:** 2019-09-20

**Authors:** Yucel Aydin, Ramazan Kurt, Kyoungsub Song, Dong Lin, Hanadi Osman, Brady Youngquist, John W. Scott, Nathan J. Shores, Paul Thevenot, Ari Cohen, Srikanta Dash

**Affiliations:** 1 Department of Pathology and Laboratory Medicine, Tulane University Health Sciences Center, New Orleans, LA 70112, USA; yaydin@tulane.edu (Y.A.); ksong@tulane.edu (K.S.); dlin6@tulane.edu (D.L.); hosman1@tulane.edu (H.O.); byoungquist@tulane.edu (B.Y.); jscottmd@tulane.edu (J.W.S.); 2 Section of Gastroenterology and Hepatology, Tulane University Health Sciences Center, New Orleans, LA 70112, USA; rkurt@tulane.edu (R.K.); shoresnj@gmail.com (N.J.S.); 3 Liver Transplant Surgery Section, Ochsner Medical Center, New Orleans, LA 70121, USA; paul.thevenot@oschner.org (P.T.); acohen@oschner.org (A.C.)

**Keywords:** hepatitis C virus (HCV), cirrhosis, hepatocellular carcinoma (HCC), endoplasmic reticulum (ER) stress, oxidative stress (OS), unfolded protein response (UPR), *microRNA-122* (*miR-122*), nuclear factor erythroid 2-related factor 2 (NRF2), signal transducer and activator of transcription 3 (STAT3), hepatocyte nuclear factor 4 alpha (HNF4A)

## Abstract

Hepatitis C virus (HCV) infection compromises the natural defense mechanisms of the liver leading to a progressive end stage disease such as cirrhosis and hepatocellular carcinoma (HCC). The hepatic stress response generated due to viral replication in the endoplasmic reticulum (ER) undergoes a stepwise transition from adaptive to pro-survival signaling to improve host cell survival and liver disease progression. The minute details of hepatic pro-survival unfolded protein response (UPR) signaling that contribute to HCC development in cirrhosis are unknown. This study shows that the UPR sensor, the protein kinase RNA-like ER kinase (PERK), mediates the pro-survival signaling through nuclear factor erythroid 2-related factor 2 (NRF2)-mediated signal transducer and activator of transcription 3 (STAT3) activation in a persistent HCV infection model of Huh-7.5 liver cells. The NRF2-mediated STAT3 activation in persistently infected HCV cell culture model resulted in the decreased expression of hepatocyte nuclear factor 4 alpha (HNF4A), a major liver-specific transcription factor. The stress-induced inhibition of HNF4A expression resulted in a significant reduction of liver-specific *microRNA-122* (*miR-122*) transcription. It was found that the reversal of hepatic adaptive pro-survival signaling and restoration of *miR-122* level was more efficient by interferon (IFN)-based antiviral treatment than direct-acting antivirals (DAAs). To test whether *miR-122* levels could be utilized as a biomarker of hepatic adaptive stress response in HCV infection, serum *miR-122* level was measured among healthy controls, and chronic HCV patients with or without cirrhosis. Our data show that serum *miR-122* expression level remained undetectable in most of the patients with cirrhosis (stage IV fibrosis), suggesting that the pro-survival UPR signaling increases the risk of HCC through STAT3-mediated suppression of *miR-122*. In conclusion, our data indicate that hepatic pro-survival UPR signaling suppresses the liver-specific HNF4A and its downstream target *miR-122* in cirrhosis. These results provide an explanation as to why cirrhosis is a risk factor for the development of HCC in chronic HCV infection.

## 1. Introduction

Hepatocellular Carcinoma (HCC) is the most common form of liver cancer. The incidence of HCC has nearly doubled over the past decade, making it the third-leading cause of cancer-related death worldwide [1]. In most cases, HCC develops on the background of cirrhosis that is mainly associated with viral hepatitis, overconsumption of alcohol, and non-alcoholic fatty liver disease. In the USA, chronic hepatitis C virus (HCV) infection is the leading cause of HCC and liver transplantation [2]. Globally, 30% of all HCC cases are related with persistent HCV infection representing a significant public health problem [3]. Recently approved direct-acting antivirals (DAAs) enable to cure the majority of patients with chronic HCV infection, including patients with cirrhosis [4]. HCV treatment reduces liver inflammation, fibrosis, and HCC thus drops liver-disease related mortality in the future [5,6]. Although the causal relationship between HCV infection and chronic liver disease has been well documented, the minute mechanisms of the virus-cell interactions involved in disease progression are not well understood. Since there is no effective treatment option of cirrhosis and late-stage HCC, it is hoped that understanding the mechanisms of HCV-induced liver cancer will allow developing better treatment. This knowledge will also result in a better understanding of the pathogenesis of other agents such as hepatitis B virus (HBV) and non-viral agents leading to cirrhosis and HCC.

The mechanisms of how chronic HCV infections are associated with the development of cirrhosis and HCC have been a challenge to study due to lack of an appropriate small animal model [7]. In a previous publication, it was shown that hepatocytes develop an integrated stress response (ISR) due to the combination of endoplasmic reticulum (ER) and oxidative stress occurring with persistent HCV replication in the cell culture model [8]. The increased cellular stress response activates nuclear factor erythroid 2-related factor 2 (NRF2) signaling as a primary cell survival pathway by inducing the transcription of genes with the antioxidant response [9]. Previously published data have shown that the stress-induced NRF2 signaling induces expression of LAMP2A and HSC70 leading to induction of chaperone-mediated autophagy (CMA). The CMA induction in HCV-infected culture improves cell survival through degradation of major tumor suppressors: p53 and retinoblastoma protein (pRB) [10,11,12]. A recent publication from this laboratory has shown that HCV-mediated NRF2 signaling impairs autophagy process at the level of autophagosome-lysosome fusion leading to activation of the oncogenic signaling through the tyrosine kinase receptors (EGFR) [13]. These data support that the activation of NRF2 signaling contributes to cell survival and progression of preneoplastic lesion to HCC malignancies [14]. Signal transducer and activator of transcription 3 (STAT3) pathway is also activated as a major cell survival mechanism in HCC but not in the surrounding non-tumor tissue or normal liver [15,16]. The mechanisms of STAT3 activation could be due to an elevated expression of cytokines such as interleukin 6 (IL6), interleukin 22 (IL22), oxidative stress or epigenetic regulation [17,18]. However, studies have shown that STAT3 activation is transient in non-transformed cells even in the presence of cytokines and STAT3 activating mutations are rarely present in HCC [17,18]. The HCC-specific mechanism of STAT3 activation has not been addressed thoroughly. 

Due to these reasons, we investigated the contribution of NRF2-related pro-survival mechanism through STAT3 activation in HCV culture in response to oxidative stress. We investigated STAT3-mediated pro-survival mechanism via a circuit that involves downregulation of hepatocyte nuclear factor 4 alpha (HNF4A) through *miR-24* and *miR-619* [19,20]. STAT3-inducible *miR-24* and *miR-629* destabilize the *HNF4A* mRNA leading to the permanent suppression of protein expression. HNF4A is a principal transcription factor required for liver development, hepatocyte differentiation, and hepatic function [21]. Many recent publications claim that the expression level of HNF4A and its target genes are impaired in cirrhosis and diminished in HCC [22,23,24,25,26,27]. HNF4A has been shown to play a role in HCC development related to chronic inflammation processes of the liver through regulating the transcription of *miR-122* [28,29,30]. The importance of this circuit in HCC development is further supported by previous reports showing that *miR-122* knockout mice develop HCC [31,32,33]. Based on these evidences, we hypothesized that the NRF2-mediated activation of the STAT3-HNF4A inflammatory loop could lead to a long-term suppression of *miR-122* that increases the HCC risk among patients with cirrhosis.

Using a persistently HCV-infected Huh-7.5 liver cell culture model, it was found that the hepatic adaptive response through the protein kinase RNA-like ER kinase (PERK)-NRF2 axis activates the STAT3-HNF4A inflammatory loop as a cell survival mechanism. Data presented in this manuscript suggest that the NRF2-mediated STAT3 activation silence the expression of HNF4A through *miR-24* upregulation. The activation of the STAT3-HNF4A loop leads to suppression of *miR-122* in persistently infected HCV cell culture and chronic HCV patients with cirrhosis. Finally, the data in the study demonstrate that the serum *miR-122* levels are depleted during advanced liver disease such as cirrhosis, which may explain why HCC develops most of the time on the background of chronic liver disease with cirrhosis.

## 2. Results

### 2.1. Persistent HCV Replication Leads to ER Stress and Oxidative Stress

To understand the hepatic adaptive response to ER stress, highly permissive Huh-7.5 cells were infected with JFH-AM120 at a multiplicity of infection (MOI) of 0.1, and HCV replication was studied over 21 days with a regular passage of infected cells at three-day intervals. Infected cells collected at different time points were examined for HCV protein expression by Western blotting. The efficiency of replication and spread of HCV-green fluorescence protein (GFP) chimera virus were examined by fluorescence microscopy with nuclear DAPI staining. Quantifications of GFP positive cells by ImageJ software show that the level of HCV replication increased with time. The percentage of Huh-7.5 cells expressing HCV-GFP fusion was quantified by flow cytometry analysis at 0, 3, 6, 9, and 12 days. These results indicated that about 5.9% of cells were GFP positive on day 3, and the number increased to 85.4% and 93.5% on day 9 and day 12, respectively. All these results support our previously published data showing that the high-level replication and spread of JFH-AM120 chimera HCV clone in Huh-7.5 liver cells (Figure S1). When the accumulation of viral protein exceeds the folding capacity of the ER resident chaperones, it creates a stress response called UPR. The activation of UPR secondary to ER stress response is the major driver of liver disease progression in HCV infection. Sustained HCV infection in Huh-7.5 liver cells is expected to accumulate unfolded protein load and expansion of the ER membranes [34]. We measured misfolded protein stress in the ER membranes by live cell imaging using a transient transfection-based approach with a commercially available ER-red fluorescence protein (RFP) construct (ER-RFP, BacMam 2.0, ThermoFisher, Waltham, MA, USA). The staining was intense in the rough ER around the nucleus of infected cells and the RFP signal overlapped with HCV-GFP. The ER staining of the uninfected cells was diffuse, whereas it was more intense in HCV-infected cells, suggesting the evidence of ER membrane expansion and accumulation of ER-RFP protein in the cells replicating HCV. Interestingly, HCV infection induced an extensive colocalization of ER-Tracker RFP with NS5A-GFP. The yellow fluorescence signal due to colocalization was markedly increased after overlaying the images of NS5A-GFP and ER-tracker RFP fluorescence. Quantitative assessment of the colocalization of HCV-GFP with ER-tracker RFP was significantly higher in HCV-infected cells than uninfected Huh-7.5 cells. These data confirm that HCV-GFP fusion protein is colocalized with the RFP protein in the perinuclear region of the infected cells that is consistent with increased GRP78/94 protein localization in the ER [35,36,37]. These results are also consistent with previous reports confirming the activation of the stress sensor of the UPR by Western blot analysis [13]. During HCV replication, several reactive oxidants such as reactive oxygen species (ROS) or reactive nitrogen species (RNS) are generated as a by-product of biochemical reactions in mitochondria, peroxisomes, and ER membranes leading to oxidative stress. A fluorescent-based assay was used to quantify ROS levels between uninfected and persistently infected cultures with HCV-Renilla luciferase virus (JFH1-V3-Rluc) on day 9. In this assay, H2DCFDA is converted to the highly fluorescent 2’7’-dichlorofluorescein due to the presence of ROS. The fluorescence intensity is proportional to the amount of oxidant present in HCV-infected cells. A flow cytometric analysis based on the quantitative approach displays that the majority of cells infected with HCV show punctate fluorescence staining due to the presence of high oxidative stress response. The oxidative stress was significantly higher in HCV-infected culture than uninfected Huh-7.5 cells by three repeated analyses. Results shown in Figure S2 confirm previously published data indicating that HCV infection also induces ER stress and oxidative stress [38,39,40,41]. 

### 2.2. Oxidative Stress and ER Stress Activate NRF2 Signaling in HCV Infection

A transcription factor, NRF2, plays a critical role in the control of genes involved in cell survival pathway. It induces varieties of cytoprotective genes harboring a short cis-acting sequence called the antioxidant response element (ARE) in their promoters [42,43]. First, we demonstrated that robust expression of the HCV core protein in the infected culture could be detected on day 3 and the number of positive cells was increased from day 6 and over 90% cells became core positive on day 12. The intensity of cytoplasmic core staining was quantified by ImageJ software (NIH, Bethesda, MD, USA) and found to increase over time, indicating active viral replication and spread after HCV infection. The activation of NRF2 signaling was examined by measuring the nuclear translocation in persistently infected culture in a kinetic study by immunocytochemical staining. NRF2 nuclear accumulation was found in 100% of HCV-infected culture starting from day 9 that correlated well with HCV core expression (Figure S3). These results indicate that HCV infection results in NRF2 activation and nuclear translocation. 

### 2.3. Persistent HCV Infection Activates the NRF2-STAT3-HNF4A Regulatory Axis as a Cytoprotective Response

**Figure 1:**
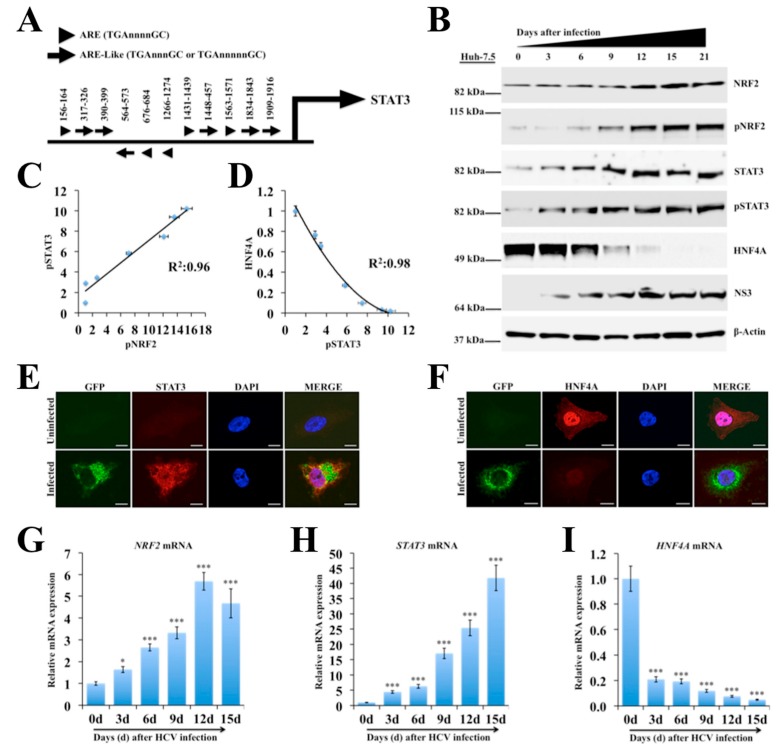
Persistent hepatitis C virus (HCV) replication induces nuclear factor erythroid 2-related factor 2 (NRF2)-mediated regulation of the signal transducer and activator of transcription 3 (STAT3)-hepatocyte nuclear factor 4 alpha (HNF4A) inflammatory loop. (**A**) STAT3-promoter region was examined for the presence of antioxidant response element (ARE) consensus sequences indicated by arrowheads, and ARE-like sequences shown by arrows. (**B**) Western blot analysis shows expression levels of total and phosphorylated NRF2, and STAT3 in infected Huh-7.5 liver cells over 21 days. The expression of HNF4A was inversely correlated with HCV NS3 protein expression. (**C**) Band intensities of phosphorylated STAT3 (pSTAT3) and phosphorylated NRF2 (pNRF2) were quantified using ImageJ software and R^2^ values were determined by excel software. (**D**) Western blot results of HNF4A and pSTAT3 were quantified using ImageJ software and R^2^ values were determined by excel software. (**E**) Colocalization of HCV-green fluorescence protein (GFP) with STAT3 by confocal microscopy. (**F**) Colocalization studies by confocal microscopy between HCV-GFP and nuclear expression of HNF4A in infected Huh-7.5 cells on day 9. Messenger RNA (mRNA) levels of individual genes in HCV-infected Huh-7.5 culture were measured by real-time RT-PCR analysis. (**G**) *NRF2* mRNA levels (**H**) *STAT3* mRNA levels. (**I**) *HNF4A* mRNA levels. The results are expressed as the mean ± standard deviation (SD) of three experiments. Error bars represent SD. *p*-values were calculated by ANOVA as compared to uninfected control. * *p*-value < 0.05, *** *p*-value < 0.001. Original magnification ×60. Scale bars = 10 μm.

**Figure 2:**
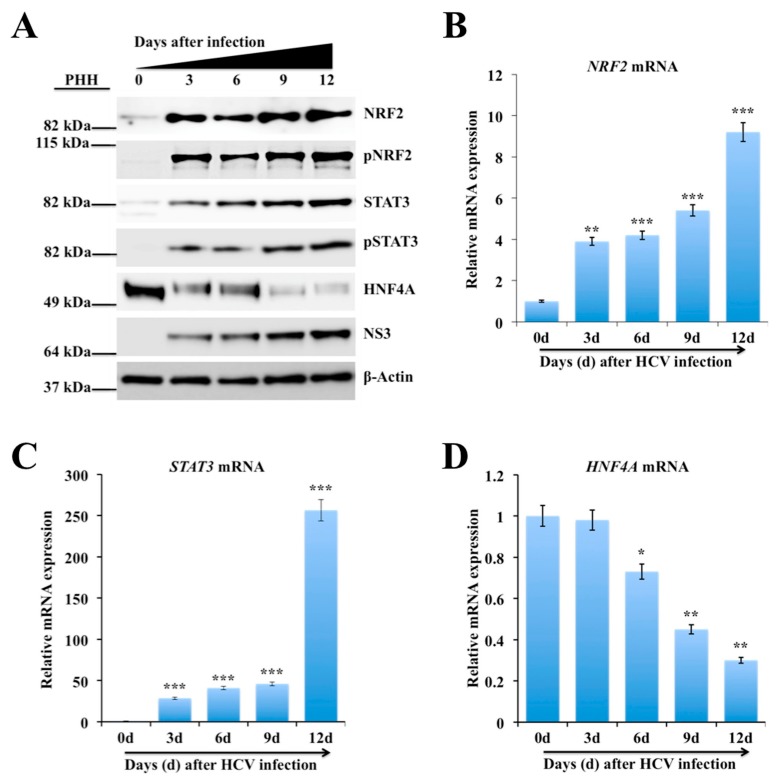
Hepatic stress response due to hepatitis C virus (HCV) replication in non-proliferative primary human hepatocytes (PHHs) activates nuclear factor erythroid 2-related factor 2 (NRF2)-mediated signal transducer and activator of transcription 3 (STAT3)-hepatocyte nuclear factor 4 alpha (HNF4A) expression. (**A**) Cell lysate was examined for the presence of HCV NS3 protein and for the activation of NRF2, STAT3 and HNF4A protein expression in infected PHH by Western blot. Messenger RNA (mRNA) levels of individual genes in HCV-infected PHHs were measured by real-time RT-PCR analysis. (**B**) Shown are *NRF2* mRNA levels (**C**) Shown are *STAT3* mRNA levels. (**D**) Shown are *HNF4A* mRNA levels. The results are expressed as the mean ± standard deviation (SD) of three experiments. Error bars represent SD. *p*-values were calculated by ANOVA as compared to day 0. * *p*-value < 0.05, ** *p*-value < 0.01, *** *p*-value < 0.001.

Increased oxidative stress has been reported in liver disease caused by viral hepatitis and non-viral etiologies [43]. The NRF2 has a crucial role in enabling adaptation to oxidative stress and ER stress by transcribing more than 2000 cytoprotective genes involved in cell survival [44,45]. The induction of NRF2 genes requires a common NRF2-binding motif on the promoter called ARE [46,47]. Five ARE (TGnnnnGC) and six ARE-like (TGAnnnnGC or TGAnnnnnGC) binding sites were identified in STAT3 promoter region (Figure 1A). Western blot analysis using phosphorylated and unphosphorylated specific antibodies revealed that HCV infection induced NRF2 and STAT3 activation (Figure 1B). The expression of STAT3 was found to be increased more in HCV-infected culture as compared to uninfected Huh-7.5 cells. The expression level of β-Actin did not change, suggesting that the differences were not at the level of protein loading or variation in the protein content of the lysates used. The inflammatory circuit consisting of HNF4A, *miR-122*, IL6, STAT3, and *miR-24* is implicated in hepatocellular transformation and liver oncogenesis [19]. The first component of the circuit is the STAT3-mediated suppression of HNF4A through *miR-24*. The second component is HNF4A-mediated suppression of *miR-122* transcription. Western blot analysis was performed to verify whether HCV-induced STAT3 also modulates expression of HNF4A pathway. Results shown in Figure 1B demonstrate that HCV infection increased NRF2, and STAT3 expression but suppressed HNF4A expression. Western blot data were quantified using ImageJ software to compare relative expression of NRF2, STAT3 and HNF4A (Figure S4). To determine the statistical correlation coefficient between the expression levels of STAT3 and HNF4A, the fraction of variance denoted by R^2^ values was calculated (Figure 1C,D). Analyses of the R^2^ values are very close to 1.0, suggesting that the induction of STAT3 correctly predicts the decrease of HNF4A in HCV-infected cells. Immunolocalization of STAT3 and HNF4A was performed in uninfected and infected cell culture on day 9 by confocal microscopy. This analysis showed that STAT3 activation in HCV-infected culture was associated with negative HNF4A expression in the nucleus (Figure 1E,F). These results indicate an inverse relationship between STAT3 activation and the expression of liver-specific transcription factor, HNF4A. Real-time RT-PCR data showed that HCV infection induced *NRF2* and *STAT3* mRNA levels, whereas *HNF4A* mRNA levels were decreased (Figure 1G–I), suggesting that activation of NRF2 signaling decreases the HNF4A protein level by altering the stability or reduced transcription of HNF4A. These results are consistent with the previous report suggesting that HCV infection inhibits *HNF4A* expression by reducing its mRNA levels [48]. To test the consistency of this observation, results were verified using an HCV infection model of primary human hepatocytes (PHHs). It was found that the expression of total NRF2 and phosphorylated NRF2 (pNRF2) were induced in HCV-infected PHHs by Western blot analysis measured for 12 days (Figure 2A). It was observed that the expression of total STAT3 and phosphorylated STAT3 (pSTAT3) were increased in a time-dependent manner in the infected PHHs culture as compared to uninfected PHHs, indicating that HCV replication induces STAT3 pathway. The expression level of HNF4A was decreased after HCV infection and inversely correlated with expression of HCV NS3 by Western blot analysis. All the Western blot bands were quantified using ImageJ software to compare the relative expression of NRF2, STAT3 and HNF4A (Figure S5). Total RNA isolated from infected PHHs was used to quantify the mRNA levels of STAT3, NRF2, HNF4A and internal control β-Actin by real-time RT-PCR. First, Ct values of individual gene were normalized with *β-Actin* mRNA and then, the fold change was determined as compared to uninfected PHHs. The mRNA levels of NRF2 and STAT3 were found to be increased in a time-dependent manner in HCV-infected PHHs (Figure 2B,C), whereas *HNF4A* mRNA levels were decreased over time post infection (Figure 2D). 

### 2.4. Silencing PERK and NRF2 Pathway Restore Expression of STAT3-HNF4A Inflammatory Loop

**Figure 3:**
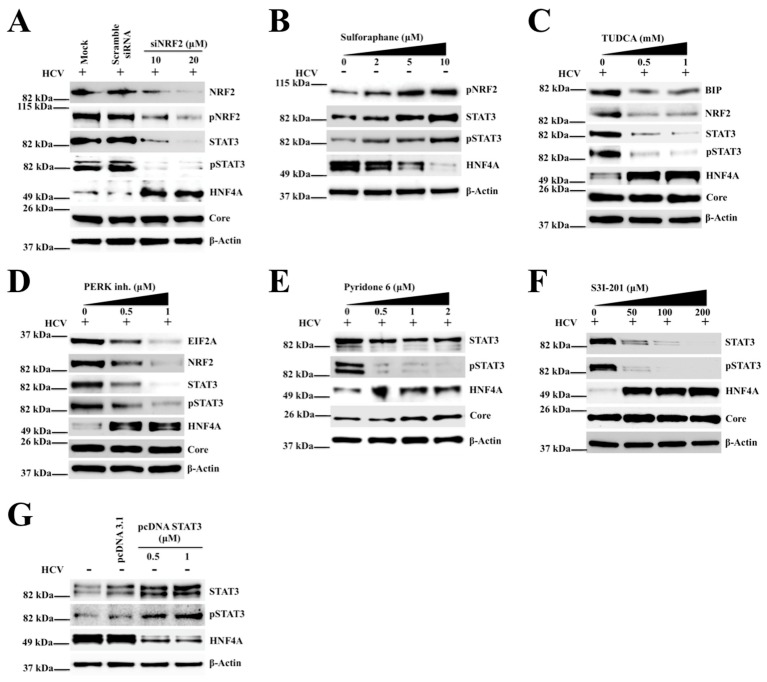
Shown is the nuclear factor erythroid 2-related factor 2 (NRF2)-dependent regulation of signal transducer and activator of transcription 3 (STAT3)-hepatocyte nuclear factor 4 alpha (HNF4A) expression in hepatitis C infection (HCV) infection. (**A**) The effect of NRF2 silencing on the expression of STAT3-HNF4A in HCV culture. Infected Huh-7.5 cells on day 9 were treated with combination of two small interfering RNAs (siRNAs) targeted to NRF2 and scrambled siRNA and the expression of STAT3 and HNF4A was measured by Western blot analysis after 72 hours. The effect of siRNA transfection did not alter HCV core expression or expression of β-Actin levels. (**B**) Shown is the effect of treatment with the NRF2 activator, sulforaphane, on the expression of STAT3 and HNF4A in uninfected Huh-7.5 liver cells. (**C)** Western blot analysis shows the effect of the endoplasmic reticulum (ER) stress inhibitor (TUDCA) on the expression levels of STAT3 and HNF4A in HCV-infected culture. (**D**) Shown are the expression levels of STAT3-HNF4A in HCV culture treated with the protein kinase RNA-like ER kinase (PERK) inhibitor by Western blot analysis. (**E**) Shown is the effect of the Janus kinase (JAK) inhibitor on the phosphorylation of STAT3 and HNF4A regulation. (**F**) Shown is the effect of the STAT3 inhibitor on the expression levels of HNF4A in infected cells by Western blot analysis. (**G**) The effect of STAT3 over expression by plasmid transfection on the levels of HNF4A in uninfected Huh-7.5 cells.

Persistent HCV replication induces sustained nuclear translocation of NRF2 as a cytoprotective mechanism in response to ER stress and oxidative stress. The impact of silencing NRF2 by a small interfering RNA (siRNA) treatment on the expression of STAT3 and HNF4A was examined in a persistently infected HCV cell culture model. The siRNA treatment was performed using day 9 infected HCV-GFP culture. As shown in Figure 3A, knockdown of NRF2 by siRNA decreased STAT3 expression and restored HNF4A expression indicating that the increased NRF2-mediated STAT3 expression is inversely associated with HNF4A downregulation. Uninfected Huh-7.5 cells treated with the NRF2 activator (sulforaphane) shows increased expression of phosphorylated NRF2 and STAT3 but suppressed HNF4A expression (Figure 3B). These data suggest that NRF2 mediated the regulation of the STAT3-HNF4A inflammatory loop in HCV infection. The relationship of NRF2 activation and ER stress was determined in infected Huh-7.5 cells after treatment with the ER stress inhibitor. As shown in Figure 3C, tauroursodeoxycholic acid (TUDCA) treatment decreased NRF2 expression, and STAT3 activation but restored HNF4A expression. To test the role of the PERK pathway on regulating the STAT3-HNF4A axis, the expression levels of STAT3 and HNF4A were measured after treatment with the PERK inhibitor by Western blotting. As shown in Figure 3D, inhibiting PERK blocked STAT3 induction and restored HNF4A expression. It is well known that STAT3 is phosphorylated by Janus kinases (JAK) leading to homo- or heterodimers, and nuclear translocation for transcriptional regulation of microRNAs and numerous genes involved in cell survival pathway. We examined whether treatment with the JAK inhibitor could also prevent STAT3 phosphorylation and restore HNF4A expression in HCV-infected culture. Day 9 infected Huh-7.5 cell culture was treated with increasing concentrations of the JAK inhibitor (pyridone 6, Calbiochem, San Diego, CA, USA) and after 72 hours, the expression of STAT3, pSTAT3 and HNF4A levels was examined by Western blotting (Figure 3E). Treatment with the JAK inhibitor did not block HCV replication but prevented STAT3 phosphorylation and restored expression of HNF4A. Furthermore, we found that infected Huh-7.5 cells treated with a highly specific STAT3 inhibitor (S3I-201, Selleck Chemicals, Houston, TX, USA) restored the expression level of HNF4A without altering level of HCV core expression (Figure 3F). Over expression of STAT3 by transient transfection downregulated the expression of HNF4A (Figure 3G). Huh-7.5 cells transfected with a control plasmid did not alter expression of HNF4A, suggesting specific regulation by STAT3 expression. The relative expression of NRF2, STAT3 and HNF4A under different siRNA or chemical treatments was compared by quantifying the Western blot results by ImageJ software (Figure S6). 

### 2.5. Persistent HCV Replication in Huh-7.5 Cells Leads to STAT3-HNF4A-Mediated Silencing of miR-122

**Figure 4:**
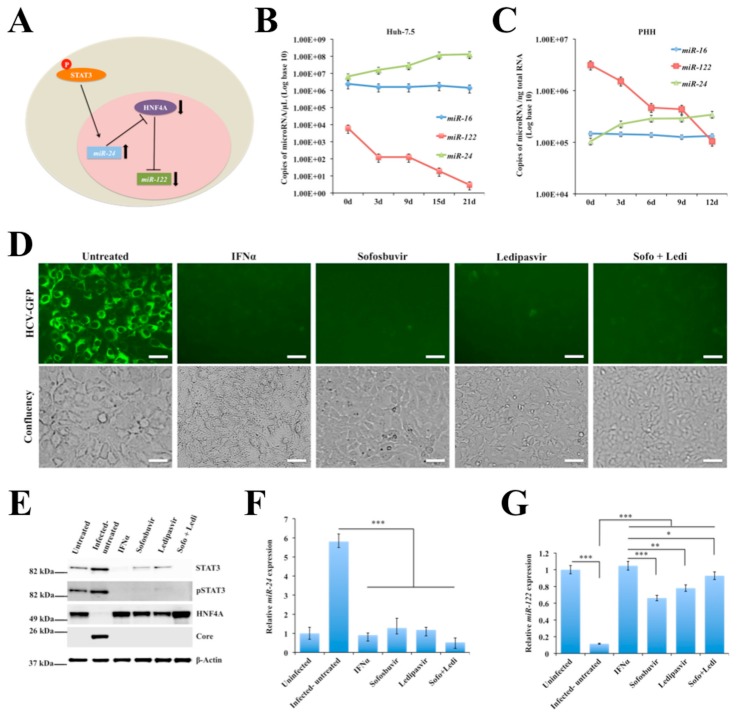
Relationship between signal transducer and activator of transcription 3 (STAT3)-hepatocyte nuclear factor 4 alpha (HNF4A) and the expression *miR-122* and *miR-24* in hepatitis C virus (HCV)-infected Huh-7.5 cells and primary human hepatocytes (PHHs). (**A**) Shown is the proposed hypothesis relating stress and STAT3-*miR-24*-HNF4A-*miR-122* feedback circuit in HCV infection. (**B**) Quantification of *miR-16*, *miR-24* and *miR-122* levels in persistently HCV-infected Huh-7.5 cells by real-time RT-PCR analysis over 21 days. (**C**) Quantification of *miR-122*, *miR-24* and *miR-122* levels in HCV-infected PHHs over 12 days. (**D**) Shown is the HCV-green fluorescence protein (GFP) expression in the infected culture with or without antiviral treatment. (**E**) Western blot shows the expression levels of STAT3 and HNF4A in the infected culture on day 9 with or without antiviral treatment. (**F**) The expression levels of *miR-24* in HCV-infected culture before and after treatment with interferon alpha (IFNα) or direct-acting antivirals (DAAs). (**G**) The expression levels of *miR-122* in HCV-infected culture treated with IFNα or DAA. The results are expressed as the mean ± standard deviation (SD) of three experiments. Error bars represent SD. *p*-values were calculated by ANOVA between different groups as compared to untreated HCV-infected group. * *p*-value < 0.05, ** *p*-value < 0.01, *** *p*-value < 0.001. Original magnification ×40, scale bars = 25 μm.

STAT3-inducible *miR-24* controls the stability of *HNF4A* mRNA through binding to the 3’untranslated region (3’UTR). This regulation is linked to HNF4A-mediated induction of liver-specific *miR-122* transcription [49]. We tested the hypothesis whether NRF2-mediated cellular adaptive response to HCV infection regulates the STAT3-HNF4A inflammatory loop through modulating the expression of *miR-122* and *miR-24* (Figure 4A). We used a RT-PCR assay to measure their expression in persistently infected culture over 21 days. As expected, the *miR-24* expression progressively increased whereas the copy number of *miR-122* decreased in HCV culture over time (Figure 4B). We found that HCV-induced stress response decreased the expression of *miR-122* in a time-dependent manner. The appearance of control unrelated *miR-16* did not change under similar assay conditions. The impact of HCV replication on the regulation of microRNA loop was also verified in infected PHHs model. It was observed that *miR-122* level decreased in HCV-infected PHHs whereas *miR-24* level increased (Figure 4C). The level of *miR-16* did not change due to HCV infection in PHHs. We conducted as a next step a series of antiviral treatment using HCV-infected Huh-7.5 cells on day 9 to see whether HCV clearance could reverse the expression of *miR-122*. As shown in Figure 4D, IFN alpha (IFNα), sofosbuvir, ledipasvir and a combination of sofosbuvir and ledipasvir effectively cleared HCV-GFP expression without affecting viability of infected Huh-7.5 cells. Furthermore, it was found that inducing HCV clearance by treatment with either IFNα or DAAs (sofosbuvir, and ledipasvir) inhibited STAT3 protein levels and restored HNF4A expression (Figure 4E). The relative expression of STAT3, HNF4A and HCV core was determined quantifying the Western blot results by ImageJ software (Figure S7). The cross-regulatory effect of STAT3 and HNF4A expression on *miR-24* and *miR-122* levels was also verified after HCV clearance with different antivirals. It was found that restoration of *miR-122* level was more efficient by IFN-based antiviral treatment than by DAAs (Figure 4F,G). These results suggest that cellular adaptive response to HCV-induced stress activated the STAT3-HNF4A inflammatory loop that leads to decreased expression of *miR-122*. 

**Figure 5:**
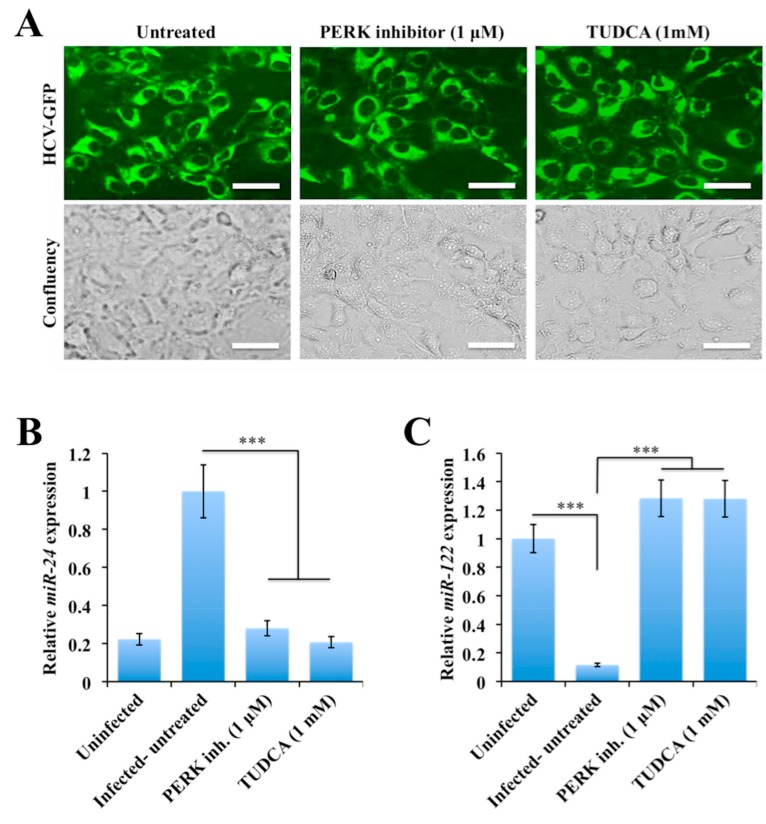
The expression of *miR-24* and *miR-122* in hepatitis C virus (HCV)-infected Huh-7.5 cells in the presence of the protein kinase RNA-like ER kinase (PERK) or endoplasmic reticulum (ER) stress inhibitors. Infected Huh-7.5 cells were treated with the PERK inhibitor or ER stress inhibitors for 72 hours. Cells were collected and the expression levels of *miR-24* and *miR-122* were measured by real-time RT-PCR. (**A**) Shown are expression levels of HCV-green fluorescence protein (GFP) chimera in day 9 HCV culture by fluorescence microscopy before and after treatment. (**B**) Shown are amounts of *miR-24* before and after treatment with the PERK inhibitor or tauroursodeoxycholic (TUDCA) in HCV-infected culture. (**C**) The levels of *miR-122* in uninfected and infected Huh-7.5 cells treated with either with the PERK inhibitor or TUDCA. The results are expressed as the mean ± standard deviation (SD) of three experiments. Error bars represent SD. *p*-values were calculated by ANOVA between different groups as compared to untreated HCV-infected group. *** *p*-value < 0.001. Original magnification ×40. Scale bars = 25 μm.

It was also of interest to determine if the treatment of HCV-infected cells with small molecule inhibitors of PERK and ER stress could show a differential effect on the expression of microRNA that controls the expression of STAT3 and HNF4A. Infected Huh-7.5 cells on day 9 was treated with a PERK inhibitor or TUDCA for 72 hours and then the expression levels of *miR-24* and *miR-122* were examined by real-time RT-PCR. Data shown in Figure 5A indicate that this treatment did not inhibit HCV replication since no difference of HCV-GFP expression between untreated and treated groups was seen. The expression level of *miR-24* was higher in HCV-infected untreated culture due to STAT3 activation (Figure 5B). As expected, persistently infected HCV culture treated with the PERK inhibitor and TUDCA inhibited *miR-24* expression but restored expression of *miR-122* in HCV culture (Figure 5B,C). Taken together, these results suggested that HCV-induced PERK activation promoted the activation of the STAT3-HNF4A inflammatory loop to overcome the stress response associated with persistent HCV replication.

### 2.6. Persistent HCV Replication in Huh-7.5 Cells Leads to Decreased miR-122 Promoter Activity

**Figure 6:**
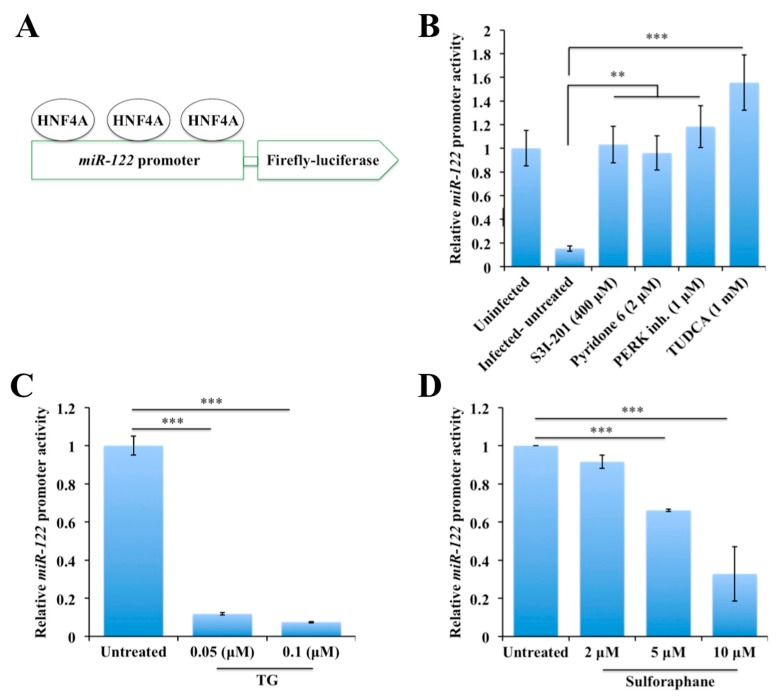
The impact of endoplasmic reticulum (ER) stress on the *miR-122* promoter activity. (**A**) There are three hepatocyte nuclear factor 4 alpha (HNF4A) binding sites present in *miR-122* promoter, which explain why HNF4A downregulation decreases *miR-122* under stress. (**B**) The effect of small molecule inhibitors targeted to signal transducer and activator of transcription 3 (STAT3), Janus kinase 1 (JAK1), protein kinase RNA-like ER kinase (PERK) and ER stress on the *miR-122* promoter regulation. Uninfected and hepatitis C virus (HCV)-infected Huh-7.5 cells were transfected with one microgram of *miR-122* promoter-construct with firefly luciferase. Following the transfection step, cells were treated with or without thapsigargin (TG), sulforaphane, and STAT3 inhibitors. After 24 hours, the luciferase activity was measured. Luciferase assays were performed three times. (**C**) ER stress activator regulation of *miR-122* promoter. (**D**) The impact of a nuclear factor erythroid 2-related factor 2 (NRF2) activator, sulforaphane, on *miR-122* promoter activity. The results are expressed as the mean ± standard deviation (SD) of three experiments. Error bars represent SD. *p*-values were calculated by ANOVA between different groups as compared to untreated HCV-infected group. ** *p*-value < 0.01, *** *p*-value < 0.001.

An in vitro assessment was done to study the impact of the HCV-induced stress response on *miR-122* promoter activity in the presence and absence of a stress inducer and inhibitors. A firefly luciferase reporter construct p-(5.7/3.8k) that contained the 3726 to 5645 bp region (chr18: 54263641-54265560) in the pGL3-basic vector (Figure 6A) was used to measure the impact of HCV replication on *miR-122* promoter activity [50]. First, we measured the impact of HCV replication on the promoter activity in a transient transfection assay. The infected culture on day 9 was transfected with microRNA-promoter plasmid for 24 hours, and then firefly luciferase activity was measured. The firefly activity was normalized with protein concentration. It was found that HCV infection significantly suppressed *miR-122* promoter activity in Huh-7.5 cells. The promoter activity in the HCV culture was enhanced in the presence of the STAT3 inhibitor and JAK inhibitor. The PERK inhibitor as well as the ER stress inhibitor, TUDCA, also increased *miR-122* promoter activity (Figure 6B). Second, we tested whether uninfected Huh-7.5 cells treated with the ER stress or the NRF2 activator could modulate *miR-122* promoter activity. As shown in Figure 6C, inducing ER stress in uninfected Huh-7.5 cells by thapsigargin (TG) treatment suppressed the *miR-122* promoter activity significantly. The treatment with sulforaphane also suppressed the *miR-122* promoter activity in a concentration-dependent manner (Figure 6D). All these results support the conclusion that the ER stress and the NRF2 activators actively suppressed the *miR-122* promoter activity, but ER stress inhibitors restored the HCV-induced suppression of the promoter activity.

### 2.7. Decreased Expression of miR-122 Correlates with Patients with Cirrhosis

**Figure 7:**
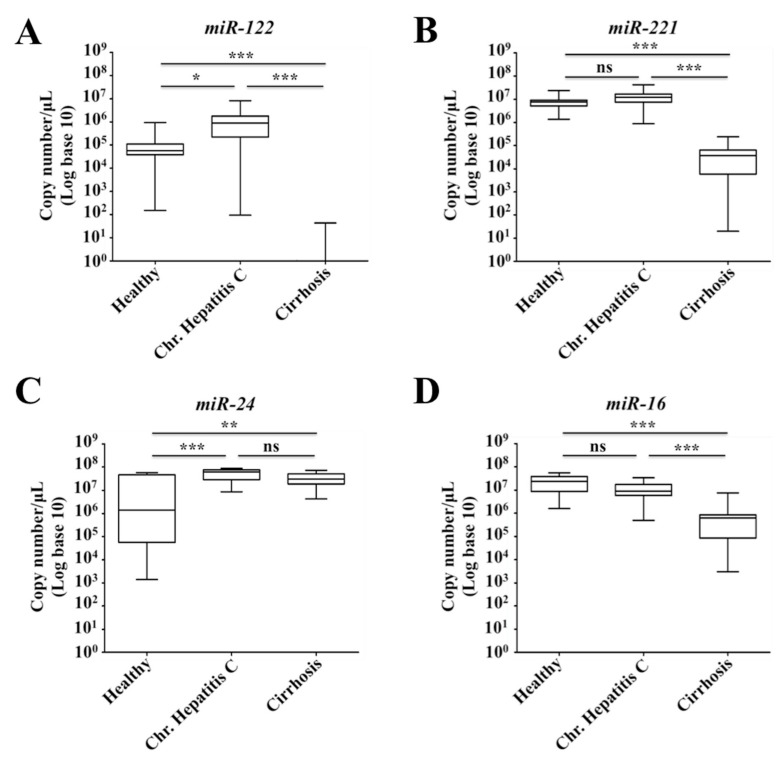
Serum levels of *miR-122*, *miR-221*, *miR-24* and *miR-16* were measured by real-time RT-PCR. (**A**) Shown are the serum *miR-122* levels among healthy individuals, chronic hepatitis C virus (HCV) infection and chronic HCV infection with cirrhosis. (**B**) Serum levels of another control liver-specific *miR-221* using the same set of serum samples. (**C**) Serum levels of signal transducer and activator of transcription 3 (STAT3)-regulated *miR-24* among healthy, chronic HCV infection and chronic HCV with cirrhosis. (**D**) Shown are the serum levels of *miR-16* in the same sets of serum samples. The results are expressed as the box plot with the median bar in triplicates. Whiskers represent minimum and maximum observed values. *p*-values were calculated by ANOVA between different groups. ns: not significant, * *p*-value < 0.05, ** *p*-value < 0.01, *** *p*-value < 0.001.

**Figure 8:**
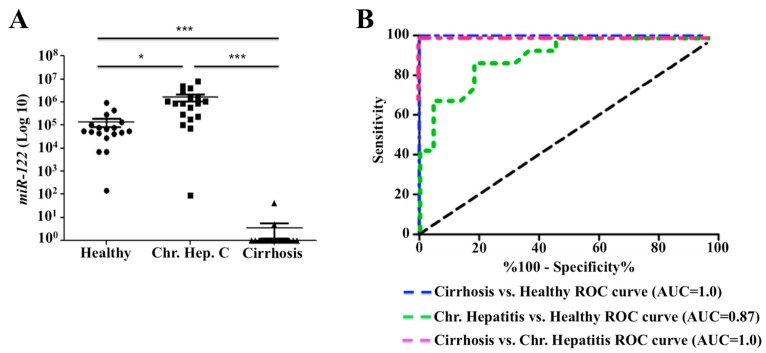
Serum *miR-122* levels discriminating patients with hepatitis C virus (HCV) infection and cirrhosis from healthy controls. (**A**) Copy number variation of serum *miR-122* levels between healthy, chronic HCV and chronic HCV with cirrhosis. (**B**) Receiver Operating Characteristic (ROC) plot. Data shown in panel A was used to generate ROC plot for determining diagnostic value of serum *miR-122* of cirrhosis and chronic HCV patients from healthy controls. The results are expressed as the mean ± standard error of mean (SEM). Error bars represent SEM. *p*-values were calculated by ANOVA between different groups. * *p*- value < 0.05, *** *p*-value < 0.001.

*MiR-122* is the most abundant liver-specific microRNA that accounts for about 70% of the total microRNA population in the adult liver [49]. Based on the fact that the cellular stress adaptation to HCV infection leads to decreased expression of *miR-122* through STAT3-HNF4A, suggesting this liver-specific microRNA may be used as a biomarker for assessing the stress response among patients with chronic HCV infection. We next sought to test this hypothesis to see whether the expression levels of *miR-122* would be different among chronically infected patient with or without cirrhosis. A retrospective analysis of serum *miR-122* levels of patients with chronic hepatitis C patients with or without cirrhosis was performed. The analysis measured the levels of *miR-122* in sera from 18 healthy, 18 chronic HCV without cirrhosis and 18 chronic HCV patients with cirrhosis samples. Total RNA was isolated from 200 μL of serum samples, and *miR-122* levels were quantified by real-time reverse transcription. Table 1 and Table 2 summarize the characteristics of the subjects included in this study. The viral titer was available in all patients with chronic hepatitis C. All cirrhotic patients were positive for HCV but viral titer was available for only 6 individuals (Table 2). It was found that the copy number of *miR-122* was higher in chronic HCV infection as compared to healthy control (*p* < 0.01). Interestingly, the *miR-122* levels were almost undetectable among most of the patients with stage IV fibrosis (Figure 7A). To rule out the possibility that decreased *miR-122* expression relates to the poor hepatic biosynthetic capability of cirrhotic liver, we measured expression of two additional microRNAs. A liver-specific *miR-221* level was measured in the same serum samples by real-time RT-PCR. These data showed that *miR-221* levels were detectable adequately (Figure 7B). The levels of STAT3-induced *miR-24* level were found to be increased in serum samples of patients with cirrhosis as compared to healthy individuals (Figure 7C). There was no difference in the serum *miR-16* level between the healthy control and chronic HCV infection without cirrhosis. The copy number of *miR-16* was comparable between normal, chronic HCV infection with or without cirrhosis (Figure 7D). There was no correlation between the age and serum *miR-122*, and *miR-16* levels, suggesting that the differences in the expression level are not related to the mean differences in the age of these patients. Serum *miR-122* levels were consistently found to be decreased since we found a significant difference between chronic HCV infection with or without cirrhosis (*p* < 0.001). Since the microRNA is required for HCV replication, we found that serum *miR-122* level was increased during chronic HCV infection. Difference in the *miR-122* level between normal healthy control and chronic HCV infection was also significant (*p* < 0.05) (Figure 8A). These data from serum testing suggest that *miR-122* expression is decreased in the advanced stage of liver disease. It was of interest to know if serum *miR-122* could be a potential diagnostic marker for cirrhosis through Receiver Operating Characteristic (ROC) plot analysis. The data showed that levels of serum *miR-122* are a potential marker for discriminating cirrhosis patients from chronic HCV and healthy control (Figure 8B). The ROC curve analysis revealed a strong separation between chronic HCV patients with cirrhosis versus without cirrhosis with an area under the curve (AUC) value 1.0, suggesting that *miR-122* is a handy marker for discriminating patients with chronic HCV from the cirrhosis group. The analysis revealed that serum levels of other *miR-16*, *miR-221* and *miR-24* were not reliable to accurately differentiate chronic HCV patients with or without cirrhosis. In summary, these results suggested that hepatic adaptive response to cellular stress associated with chronic HCV infection was stronger among the cirrhotic group that leads to depletion of serum *miR-122* level.

## 3. Discussion

Chronic liver disease associated with hepatitis virus infection (HBV and HCV) contributes to more than 70% of the HCC worldwide, whereas the remaining cases relate to non-viral etiologies [51]. Since most of HCC cases related to viral and non-viral etiology develop on the background of cirrhosis, suggesting that there may be a common pathway involved in the development of cirrhosis and HCC. However, approximately 20–35% of HCC develop without cirrhosis in the liver, suggesting that inflammation is not an absolute requirement for HCC development [52,53]. The molecular mechanism that drives the progression of cirrhosis and HCC during the chronic stage of liver disease is not understood. Whole genome sequencing found that many driver genes show altered expression in HCC related to chronic HCV infection [51]. Our hypothesis was that the ISR triggered in the infected cells as a compensatory mechanism to deal with varieties of stress responses such as oxidative stress, and ER stress during HCV replication in hepatocytes. The presence of long-lasting unresolved stress response also compromises liver function that leads to cirrhosis and HCC development. In support of this hypothesis, we showed that HCV infection induced ISR in response to ER stress and oxidative stress in cirrhosis that lead to decreased expression of type I IFN receptors [54]. Due to this reason, patients with cirrhosis frequently remain as non-responders to IFN/ribavirin-based antiviral therapy. Some of the pro-survival outcomes of the stress response are the inhibition of host protein synthesis due to increased eukaryotic translation initiation factor 2 subunit alpha (EIF2A) phosphorylation, degradation of misfolded proteins by ER-assisted protein degradation (ERAD) and autophagy. We showed that prolonged stress response activated PERK-induced NRF2 signaling as a cell survival mechanism [13]. The NRF2 signaling activates tumor-promoting autophagy (CMA) that degrades tumor suppressors and induces oncogenic signaling implicated in cell survival.

In this study, we found that NRF2 signaling activated transcription of STAT3, a member of STAT protein family, that is known to be induced by IL6 and participates in inflammation, tumorigenesis and autophagy [55]. STAT3 protein gets phosphorylated at tyrosine 705 by JAK2/tyrosine kinase 2 (TYK2), resulting from dissociation from the cytoplasmic tail of the cytokine receptors, homodimerizes and enters to the nucleus to activate gene transcriptions. Increased STAT3 activity is associated with HCC development and poor prognosis [56]. Activated STAT3 can inhibit cellular autophagy for promoting cell survival, supporting our hypothesis that prolonged stress response inhibits autophagy [57]. Other investigators and we have demonstrated that chronic inflammatory responses can activate STAT3 through IFN and IL6 production [22,58]. Although STAT3 activation occurs due to a variety of mechanisms, HCC-specific STAT3 activation mechanisms have not been well established. Our data show that cellular adaptive response to HCV-induced stress activates STAT3-mediated programing through HNF4A that leads to silencing of liver-specific *miR-122*. To support this hypothesis, we demonstrated that the excessive cellular stress associated with HCV infection activated STAT3 transcription through NRF2 signaling. Our data showed that persistent HCV infection promotes STAT3-inducible expression of *miR-24*. The increased *miR-24* transcription correlated with the decreased expression of *HNF4A* mRNA through binding to the 3’UTR in HCV infection. Our data support the data published earlier showing that an oncogenic circuit consisting of HNF4A, *miR-122*, IL6, STAT3, and *miR-24* is involved in the cell survival mechanisms under stress during HCV infection [19]. Cellular adaptive stress response associated with chronic HCV infection activated STAT3 and utilized this oncogenic circuit, which resulted in a decrease in the expression of HNF4A through *miR-24*. Nuclear HNF4A is a crucial transcription factor during embryonic liver development and hepatocyte differentiation [59]. Available literature shows that HNF4A is the major liver-specific transcription factor that modulates the expression of nearly 42% of the genes expressed in hepatocytes, involved in glucose, fatty acid metabolism, synthesis of blood coagulation factor VII, enzymes involved in hepatic detoxification, and hepatic differentiation [60,61,62,63,64,65]. Our data is supported by studies showing that mRNA and protein expression levels of HNF4A decreased severely patients with cirrhosis. Hemorrhage is a common cause of death in cirrhosis, especially variceal bleeding. Hepatocytes synthesize both clotting factors and endogenous anticoagulants. The levels of these proteins are reduced in cirrhosis. HNF4A induces the production of blood coagulation factor VII. Our study also provides an explanation why HNF4A targeted protein expression is decreased in cirrhosis due to severe hepatic stress response. Studies have shown that decreased expression of HNF4A is also correlate with bleeding disorder due to decreased expression of the blood coagulation factor VII [64,65]. 

The activation of this STAT3- HNF4A inflammatory loop led to decreased expression of *miR-124* and *miR-122*. These two microRNAs were regulated by HNF4A. This study was extended to see whether this hypothesis could be validated using prospectively collected serum samples of patients chronically infected with HCV. The measurement of serum *miR-122* levels was performed using serum samples from patients with chronic HCV infection with or without cirrhosis. It was observed that *miR-122* levels increased during the chronic stage of HCV infection, the levels significantly decreased in advanced liver disease, particularly with stage IV fibrosis called cirrhosis. Interestingly, we report here that the levels of *miR-122* have remained below the detection limit among all cirrhotic patients tested in our hand. Our study results are in agreement with a prior report showing that the serum *miR-122* level was found to decreased in liver injury in humans with advanced liver disease, including cirrhosis [66,67,68,69]. Decreased expression of *miR-122* has also been found in cirrhosis related to non-alcoholic steatohepatitis (NASH) [33]. The decreased production of *miR-122* in the cirrhotic patients was not related to the impaired biosynthetic capability of hepatocytes present in the cirrhotic liver because the expressions of a liver-specific *miR-221* and STAT3-specific *miR-24* were detectable. Our data show that unresolved stress response depletes the expression of *miR-122*, suggesting that the activation of the STAT3-HNF4A inflammatory loop also occurs in the chronic HCV infection in humans. Our findings provide two pieces of new information that may increase our understanding why STAT3 is transcriptionally activated in liver cancer. First, we show that cellular adaptive response to ER stress in HCV infection causes STAT3 activation at the level of mRNA transcription as well as phosphorylation. Second, we found that STAT3 activation is involved in silencing of *miR-122* in the cirrhosis. Recent studies have observed that epigenetic programing is involved in HCC development in cirrhosis after the viral cure [70,71,72]. We claimed that our results show a novel adaptive cell survival mechanism related to excessive ER stress that involved STAT3-mediated silencing of *miR-122* expression, the major liver-specific microRNA. The early silencing of *miR-122* and HNF4A increases the risk for HCC development in cirrhosis. 

The *miR-122* plays a central role in liver development, differentiation, hepatic metabolic function and innate immunity in the liver [66]. However, the studies investigating the regulation of *miR-122* expression during chronic liver diseases related to viral and non-viral etiologies have produced conflicting results. A handful of publications showed that the levels of *miR-122* decreased during advanced liver disease related to hepatitis C, hepatitis B, and non-viral etiologies [73,74,75]. *MiR-122* expression in the liver is critical for maintaining innate immunity and IFN production. A report by Xu et al. [73] shows that *miR-122* supports innate immunity by removing the negative regulation of STAT3 signaling on IFN expression. They showed that *miR-122* targets three receptor tyrosine kinases that directly modulate STAT3 phosphorylation. Another recent study by Van Renne et al. [74] showed that the tumor suppressor protein, tyrosine phosphatase receptor delta (PTPRD) controls the STAT3 activation in HCV-induced HCC. They found that high expression of PTPRD suppressed STAT3 phosphorylation in healthy liver tissue but low expression of this protein in HCC resulted in the tumor-specific STAT3 activation. These reports are consistent with our results that HCV infection activates STAT3. On the other hand, the liver-specific *miR-122* expression is also decreased during chronic HBV infection. Chen et al. [75] demonstrated that miR-122 binds to the highly conserved region of HBV pre genomic RNA, causing RNA degradation and reduce viral replication. Data presented in this study show that HCV replication decreased the expression of miR-122 in a time-dependent manner. Data from recent publications confirm the downregulated expression of miR-122 during HCV-induced advanced liver disease [69,76,77]. A report by Luna et al. [76] showed that HCV RNA replication occurs independent of the miR-122 interaction. The team showed that the Argonaute (AGO) protein directly binds to the miR-122 binding sites in HCV RNA, specifically sequesters *miR-122*, to repress the liver target genes during chronic HCV infection. Their investigation showed that the *miR-122* expression decreased in the liver tissues of humans and chimpanzees infected with HCV. Another report by Hyrina et al. [77] showed that the plasma level of *miR-122* decreased during chronic HCV infection and were not restored after HCV cure. Their study also showed that serum *miR-122* level was correlated with liver transaminases. These results suggest that *miR-122* could be a liver-specific biomarker after HCV cure. A study by Sarasin-Filipowicz et al. [69] reported decreased expression of *miR-122* in the liver of HCV-infected patients who were resistant to IFN-based therapy. Our data showed that severe ER stress response during high-level HCV replication in cell culture models of Huh-7.5 liver cells and PHHs decreased the expression of *miR-122*. The expression levels of *miR-122* were depleted among patients with cirrhosis, but not with chronic HCV infections. The reason why the *miR-122* level decreased only in cirrhosis, but not during chronic liver disease is unclear. The host factor requirement for HCV replication has not been fully established. It is possible that HCV replication in the cirrhotic liver may depend on factors other than *miR-122*. The potential liver regeneration and increased inflammation may contribute to increased *miR-122* level in the blood during chronic HCV infection. However, data presented in this report do not support the earlier finding showing that *miR-122* is an essential host factor for HCV replication. The *miR-122* binding does not cause HCV RNA degradation instead it stabilizes the viral genome and promotes translation [78]. In summary, the results presented in this article are consistent with many earlier publications suggesting that decreased expression of *miR-122* in cirrhosis may be associated with loss of hepatic function and innate immunity, impaired liver regeneration, as well as increased risk of HCC development.

## 4. Materials and Methods

### 4.1. Cell Culture, Antibodies and Chemicals

The highly permissive transformed liver Huh-7.5 cell line was obtained from the laboratory of Charles M. Rice (Rockefeller University, New York). The Huh-7.5 cells were cultured in Dulbecco’s Modified Eagle’s Medium (DMEM) supplemented with two mM L-glutamine, sodium pyruvate, nonessential amino acids, 100 U/mL penicillin, 100 μg/mL streptomycin, and 10% fetal bovine serum. Huh-7.5 cell culture was infected with either JFH1-GFP chimera HCV or JFH-dV3-Rluc HCV using a protocol developed in our laboratory as previously described [79]. PHHs were obtained from XenoTech LLC (Kansas City, MO, USA) and cultured with hepatocyte culture media supplemented with 10% human serum (Invitrogen, Brown Deer, WI, USA). After 24 hours they were infected with cell culture grown HCV-GFP chimera virus with a MOI of 0.1. After 18 hours of infection, hepatocytes were replaced with fresh hepatocyte culture media (XenoTech, LLC, Kansas City, MO, USA) supplemented with 10% human serum (Invitrogen, Brown Deer, WI, USA). Uninfected or infected PHH were harvested every 3 days and cell pellets were used for RNA isolation and Western blot analysis. The success of HCV replication in the infected PHHs was assessed by Western blot analysis of HCV NS3 protein. Sofosbuvir was purchased from Acme Biosciences, Inc. (Palo Alto, CA, USA), and IFNα was purchased from EMD Merck (Billerica, MA, USA). Ledipasvir and S3I-201 were obtained from Selleck Chemicals (Houston, TX, USA). TG, TUDCA, the PERK inhibitor (GSK 2606414), and sulforaphane were obtained from Sigma-Aldrich (St. Louis, CA, USA). Pyridone 6 was obtained from Calbiochem (San Diego, CA, USA). The antibody to phospho-NRF2 was purchased from Abcam (Cambridge, MA, USA). Antibodies to GRP78 (BIP), EIF2A, STAT3, phospho-STAT3, HNF4A, and β-Actin were obtained from Cell Signaling (Beverly, MA, USA). Antibody to NS3 was purchased from Virogen Inc. (Boston, MA, USA). The Antibody to HCV core was purchased from Thermo Scientific (Waltham, MA, USA). The antibody to total NRF2 was purchased from Santa Cruz Biotechnology (Santa Cruz, CA, USA).

### 4.2. Quantitative Assessment of Misfolded Protein Burden in the ER

The accumulation of misfolded proteins in the infected culture was quantified using an ER-tracker reagent called CellLight ER-RFP BacMam 2.0 (Thermo Fisher Scientific). This construct expresses Red Fluorescence Protein (RFP) fused to the ER signal sequence of calreticulin and KDEL (ER-retention signal) to quantitate the extent of red fluorescence by fluorescence microscopy and flow cytometry. This reagent allows live multiplexing imaging of ER in the cells expressing HCV-GFP fusion protein. Briefly, HCV-infected Huh-7.5 cells, and uninfected Huh-7.5 cells were incubated with ER-tracker dye overnight at 37 °C, and the next day, cells were observed using a fluorescence microscope. For nuclear staining and imaging, the cells were directly incubated with Hoechst 33342 (Thermo Fisher Scientific) at 1 μg/mL for 5 minutes in 6-well plate. Then, the cells were washed with PBS and fixed for 10 minutes with 2% paraformaldehyde. The colocalization of GFP and RFP between infected and uninfected cells quantified by flow analysis.

### 4.3. Detection of Reactive Oxygen Species (ROS)

ROS in HCV culture measured using the cell-permeant H2DCFDA reagent (Thermo Fisher Scientific) according to the manufacturer’s instructions. The 2′,7′-dichlorodihydrofluorescein diacetate (H2DCFDA) fluorescent probe reacts with ROS including hydrogen peroxide and hydroxyl radicals. The cell-permeant H2DCFDA diffuses into cells and is retained in the cytoplasm after cleavage by intracellular esterase. The ROS converts the non-fluorescent H2DCFDA to the highly fluorescent 2′,7′-dichlorofluorescein (DCF), which could be detected by fluorescent microscopy or quantified by flow cytometry. 

### 4.4. Immunohistochemical Staining

Tissue culture cells were immobilized onto a glass slide by cytospin. Immunostaining of the cytospin slides of infected cells was performed using a standard protocol established in our laboratory [11]. We used a monoclonal antibody to HCV core protein (Thermo Scientific) and monoclonal antibody that detects phosphorylated NRF2 (Abcam).

### 4.5. SDS-PAGE and Western Blotting

Infected Huh-7.5 cells were harvested by the treatment of trypsin-ethylenediaminetetraacetic acid (EDTA). Cells were lysed in ice-cold lysis buffer (50 mM Tris HCl pH 8.0, 140 mM NaCl, 1.5 mM MgCl2, 0.5% NP-40 with complete protease inhibitor from Invitrogen) for 10 minutes in ice (about 1 × 10^6^ cells/200 μL). Infected Huh-7.5 cells pelleted by low-speed centrifugation. The detergent compatible (DC) protein assay determined protein concentration. Samples were boiled for 10 minutes at 80 °C in the presence of 1× sodium dodecyl sulfate-polyacrylamide gel electrophoresis (SDS-PAGE)-loading buffer (250 mM Tris-HCl pH 6.8, 40% glycerol, 8% SDS, 0.57M β-mercaptoethanol, 0.12% bromophenol blue). Approximately 20 μg of protein was loaded onto 12% SDS-PAGE and transferred into a nitrocellulose membrane (Thermo Scientific). The membrane was blocked using a solution containing 5% of blotting-grade milk power (Bio-Rad, Hercules, CA, USA) for 2 hours then incubated with a primary antibody. After overnight incubation, the antigen-antibody complex was visualized with horseradish peroxidase (HRP)-conjugated goat anti-rabbit or anti-mouse Immunoglobulin G (IgG) and the ECL detection system (Amersham ECL, GE Healthcare Bio-Sciences, Pittsburgh, PA, USA). 

### 4.6. siRNA Transfection

Persistently HCV-infected Huh-7.5 cells were cultured in 6-well plates (up to 60% confluence in DMEM supplemented with 10% FBS media) without antibiotics. The next day, culture media was replaced with fresh DMEM with 2% FBS and cells were then transfected with siRNA to either NRF2 (siRNA1: 5′-GAAUGGUCCUAAAACACCA-3′, siRNA2: 5′-UGACAGAAGUUGACAAUUA-3′) synthesized by Invitrogen [80,81] or scramble siRNA (Invitrogen) using Lipofectamine (Life Technology, Grand Island, NY, USA). The knockdown efficiency was analyzed by Western blot. 

### 4.7. Quantification of mRNA Levels by RT-qPCR

Infected Huh-7.5 cells and PHHs were harvested at different time points post HCV infection, and total RNA was isolated using the RNeasy Mini kit (Qiagen, Germantown, MD, USA). The first strand of complementary DNA (cDNA) was synthesized from 1 μg of total RNA using an iScript Reverse Transcription Supermix (Bio-Rad). 100 ng cDNA was amplified using iTaq Universal SYBR Green Supermix (Bio-Rad) with gene-specific forward and reverse primers following instructions in the kit. The mRNA levels of NRF2, STAT3, HNF4A, and β-Actin (as internal control) were quantified using quantitative RT-PCR (RT-qPCR). Amplification, data acquisition, and analysis were carried out on the CFX96 real-time instrument using CFX manager software version 3.0 (Bio-Rad). The expression levels of mRNAs were normalized with the *β-Actin* mRNA level using the comparative threshold cycle method. The nucleotide sequences of oligonucleotide primers for *NRF2* mRNA (sense primer 5’-TACTCCCAGGTTGCCCACA-3’ and antisense primer 5’-CATCTACAAACGGGAATGTCTGC-3’) [82], for *STAT3* mRNA (sense primer 5’-TGGAAGAGGCGGCAGCAGATAGC-3’ and antisense primer 5’-CACGGCCCCCATTCCCACAT-3’) [83], for *HNF4A* mRNA (sense primer 5’-TGTCCCGACAGATCACCTC-3` and antisense primer 5’-CACTCAACGAGAACCAGCAG-3’) [20], and for *β-Actin* mRNA (sense primer 5’-GAGCTACGAGCTGCCTGAC-3’ and antisense primer 5’-AGCACTGTGTTGGCGTACAG-3’) [84] were derived from previous published reports. 

### 4.8. Quantification of Serum microRNA Levels by RT-qPCR

Total microRNA was isolated from 20 μL serum samples using miRNeasy kit (Qiagen) used for reverse transcription using miScript II RT kit (Qiagen) as described previously [85]. Individual microRNA was amplified using cDNA templates, a universal primer and a PCR kit using a recommended protocol. A standard curve was generated for determining microRNA copy number and Ct values using serially diluted synthetic microRNA. The copy number of each microRNA in the serum was calculated from the Ct values. 

### 4.9. Statistical Analysis

Western blot, immunostaining, and immunofluorescence images were quantified using a computer image analysis software package (ImageJ version 1.52c, NIH, Bethesda, MD, USA). All measurements were made at least in triplicate (*n* = 3). To compare means within groups, we performed a one-factor analysis of variance (ANOVA) using the GraphPad Prism software (GraphPad Company, San Diego, CA, USA). Data were tested and found to be normally distributed. To determine the statistical correlation coefficient between protein expressions, fraction of variance denoted by R^2^ values was determined using excel software. Sensitivity of the assays was plotted against the false positivity (1-specificity) using ROC curves using the GraphPad Prism software. Comparison of AUC was performed, which compares the AUC to the diagonal line of no information (AUC 0.5). We applied Dunnet’s post hoc test to compare control samples with experimental samples when the overall p-value for the ANOVA analysis was significant (p < 0.05). When performing comparisons between multiple groups, each analyzed with ANOVA; we used the Bonferroni correction to determine a revised cutoff for statistical significance that gives a combined 5% type I error probability. * *p*-value < 0.05, ** *p*-value < 0.01, *** *p*-value < 0.001.

## 5. Conclusions

Hepatic adaptive response to HCV-induced stress reduces liver-specific miR-122 through activation of STAT3-HNF4A inflammatory feedback loop. Serum *miR-122* could be used as a biomarker to monitor the activation of this inflammatory loop in liver cirrhosis. 

## Figures and Tables

**Table 1:** Characteristics of the patients with Chronic Hepatitis C.

Patient Number	Age	Sex	HCV RNA IU/mL	HCV Genotype	Metavir Score
1	48	F	2,000,000	1a	3
2	46	F	29,000	1b	0
3	27	F	1,000,000	1a	1.2
4	59	F	553,000	1a	3
5	64	M	2,000,000	1a	1.2
6	34	F	7,000,000	1a	1,2
7	59	F	1,000,000	2	0
8	44	M	170,000	1a	1
9	50	M	3,000,000	1b	0
10	33	F	3,000,000	1b	1.2
11	53	M	904,000	1a	1
12	62	M	7062	1a	0
13	69	F	10,000,000	1b	2
14	47	F	2,000,000	2	0
15	50	F	10,000,000	1a	0
16	48	M	2,000,000	1b	0
17	40	F	200,000	1a	1
18	53	M	815,000	1a	1

HCV: hepatitis C virus; M: male; F: female.

**Table 2:** Characteristics of the patients with cirrhosis.

Patient Number	Age	Sex	HCV RNA IU/mL	HCV Genotype	Metavir Score
1	57	M	+	1a	4
2	52	M	+	1a	4
3	60	F	+	1a	4
4	64	F	+	N/A	4
5	62	M	2,700,000	1b	4
6	66	M	+	1a	4
7	54	M	+	1a	4
8	54	M	+	1b	4
9	62	F	+	N/A	4
10	58	F	+	N/A	4
11	64	M	+	2	4
12	66	F	+	N/A	4
13	57	F	+	N/A	4
14	56	M	5,940,000	N/A	4
15	60	M	7,200,000	1a	4
16	60	M	2,860,000	1b	4
17	56	M	4,310,000	1a	4
18	60	M	6,480,000	1a	4

HCV: hepatitis C virus; M: male; F: female; N/A: Not Available.

## Supplementary Materials

The following are available online at https://www.mdpi.com/2072-6694/11/10/1407/s1, Figure S1: Persistent HCV replication model in permissive Huh-7.5 liver cells using HCV-GFP chimera virus, Figure S2: Live cell imaging of ER stress induced by HCV, Figure S3: Persistent replication of HCV-GFP chimera induces NRF2 activation, Figure S4: Quantification of Western blot of Figure 1, Figure S5: Quantification of Western blot of Figure 2A, Figure S6: Quantification of Western blot of Figure 3, Figure S7: Quantification of Western blot of Figure 4E.

## References

[1] Sayiner M., Golabi P., Younossi Z.M. (2019). Disease Burden of Hepatocellular Carcinoma: A Global Perspective. Dig. Dis. Sci..

[2] Allison R.D., Tong X., Moorman A.C., Ly K.N., Rupp L., Xu F., Gordon S.C., Holmberg S.D., Chronic Hepatitis Cohort Study (CHeCS) Investigators (2015). Increased incidence of cancer and cancer-related mortality among persons with chronic hepatitis C infection, 2006–2010. J. Hepatol..

[3] Thrift A.P., El-Serag H.B., Kanwal F. (2016). Global epidemiology and burden of HCV infection and HCV-related disease. Nat. Rev. Gastroenterol. Hepatol..

[4] Pawlotsky J.M., Feld J.J., Zeuzem S., Hoofnagle J.H. (2015). From non-A, non-B hepatitis to hepatitis C virus cure. J. Hepatol..

[5] Lombardi A., Mondelli M.U., ESCMID Study Group for Viral Hepatitis (ESGVH) (2019). Hepatitis C: Is eradication possible?. Liver Int..

[6] Thomas D.L. (2013). Global control of hepatitis C: Where challenge meets opportunity. Nat. Med..

[7] Bartosch B., Thimme R., Blum H.E., Zoulim F. (2009). Hepatitis C virus-induced hepatocarcinogenesis. J. Hepatol..

[8] Dash S., Aydin Y., Wu T. (2019). Integrated stress response in hepatitis C promotes Nrf2-related chaperone-mediated autophagy: A novel mechanism for host-microbe survival and HCC development in liver cirrhosis. Semin. Cell Dev. Biol..

[9] Raghunath A., Sundarraj K., Nagarajan R., Arfuso F., Bian J., Kumar A.P., Sethi G., Perumal E. (2018). Antioxidant response elements: Discovery, classes, regulation and potential applications. Redox Biol..

[10] Chava S., Lee C., Aydin Y., Chandra P.K., Dash A., Chedid M., Thung S.N., Moroz K., Wu T., Nayak N.C. (2017). Chaperone-mediated autophagy compensates for impaired macroautophagy in the cirrhotic liver to promote hepatocellular carcinoma. Oncotarget.

[11] Aydin Y., Chatterjee A., Chandra P.K., Chava S., Chen W., Tandon A., Dash A., Chedid M., Moehlen M.W., Regenstein F. (2017). Interferon-alpha-induced hepatitis C virus clearance restores p53 tumor suppressor more than direct-acting antivirals. Hepatol. Commun..

[12] Aydin Y., Chedid M., Chava S., Danielle Williams D., Liu S., Hagedorn C.H., Sumitran-Holgersson S., Reiss K., Moroz K., Lu H. (2017). Activation of PERK-Nrf2 oncogenic signaling promotes Mdm2-mediated Rb degradation in persistently infected HCV culture. Sci. Rep..

[13] Aydin Y., Stephens C.M., Chava S., Heidari Z., Panigrahi R., Williams D.D., Wiltz K., Bell A., Wilson W., Reiss K. (2018). Chaperone-Mediated Autophagy Promotes Beclin1 Degradation in Persistently Infected Hepatitis C Virus Cell Culture. Am. J. Pathol..

[14] Petrelli A., Perra A., Cora D., Sulas P., Menegon S., Manca C., Migliore C., Kowalik M.A., Ledda-Columbano G.M., Giordano S. (2014). MicroRNA/gene profiling unveils early molecular changes and nuclear factor erythroid related factor 2 (NRF2) activation in a rat model recapitulating human hepatocellular carcinoma (HCC). Hepatology.

[15] Yang S.F., Wang S.N., Wu C.F., Yeh Y.T., Chai C.Y., Chunag S.C., Sheen M.C., Lee K.T. (2007). Altered p-STAT3 (tyr705) expression is associated with histological grading and intratumour microvessel density in hepatocellular carcinoma. J. Clin. Pathol..

[16] He G., Yu G.Y., Temkin V., Ogata H., Kuntzen C., Sakurai T., Sieghart W., Peck-Radosavljevic M., Leffert H.L., Karin M. (2010). Hepatocyte IKKbeta/NF-kappaB inhibits tumor promotion and progression by preventing oxidative stress-driven STAT3 activation. Cancer Cell.

[17] He G., Karin M. (2011). NF-kappaB and STAT3—Key players in liver inflammation and cancer. Cell Res..

[18] Svinka J., Mikulits W., Eferl R. (2014). STAT3 in hepatocellular carcinoma: New perspectives. Hepat. Oncol..

[19] Hatziapostolou M., Polytarchou C., Aggelidou E., Drakaki A., Poultsides G.A., Jaeger S.A., Ogata H., Karin M., Struhl K., Hadzopoulou-Cladaras M. (2011). An HNF4alpha-miRNA inflammatory feedback circuit regulates hepatocellular oncogenesis. Cell.

[20] Aboulnasr F., Hazari S., Nayak S., Chandra P.K., Panigrahi R., Ferraris P., Chava S., Kurt R., Song K., Dash A. (2015). IFN-lambda Inhibits MiR-122 Transcription through a Stat3-HNF4alpha Inflammatory Feedback Loop in an IFN-alpha Resistant HCV Cell Culture System. PLoS ONE.

[21] Lau H.H., Ng N.H.J., Loo L.S.W., Jasmen J.B., Teo A.K.K. (2018). The molecular functions of hepatocyte nuclear factors—In and beyond the liver. J. Hepatol..

[22] Yue H.Y., Yin C., Hou J.L., Zeng X., Chen Y.X., Zhong W., Hu P.F., Deng X., Tan Y.X., Zhang J.P. (2010). Hepatocyte nuclear factor 4alpha attenuates hepatic fibrosis in rats. Gut.

[23] Lazarevich N.L., Cheremnova O.A., Varga E.V., Ovchinnikov D.A., Kudrjavtseva E.I., Morozova O.V., Fleishman D.I., Engelhardt N.V., Duncan S.A. (2004). Progression of HCC in mice is associated with a downregulation in the expression of hepatocyte nuclear factors. Hepatology.

[24] Lazarevich N.L., Shavochkina D.A., Fleishman D.I., Kustova I.F., Morozova O.V., Chuchuev E.S., Patyutko Y.I. (2010). Deregulation of hepatocyte nuclear factor 4 (HNF4) as a marker of epithelial tumors progression. Exp. Oncol..

[25] Ning B.F., Ding J., Yin C., Zhong W., Wu K., Zeng X., Yang W., Chen Y.X., Zhang J.P., Zhang X. (2010). Hepatocyte nuclear factor 4 alpha suppresses the development of hepatocellular carcinoma. Cancer Res..

[26] Tanaka T., Jiang S., Hotta H., Takano K., Iwanari H., Sumi K., Daigo K., Ohashi R., Sugai M., Ikegame C. (2006). Dysregulated expression of P1 and P2 promoter-driven hepatocyte nuclear factor-4alpha in the pathogenesis of human cancer. J. Pathol..

[27] Yin C., Lin Y., Zhang X., Chen Y.X., Zeng X., Yue H.Y., Hou J.L., Deng X., Zhang J.P., Han Z.G. (2008). Differentiation therapy of hepatocellular carcinoma in mice with recombinant adenovirus carrying hepatocyte nuclear factor-4alpha gene. Hepatology.

[28] Li C., Deng M., Hu J., Li X., Chen L., Ju Y., Hao J., Meng S. (2016). Chronic inflammation contributes to the development of hepatocellular carcinoma by decreasing miR-122 levels. Oncotarget.

[29] Yang Y.M., Lee C.G., Koo J.H., Kim T.H., Lee J.M., An J., Kim K.M., Kim S.G. (2015). Galpha12 overexpressed in hepatocellular carcinoma reduces microRNA-122 expression via HNF4alpha inactivation, which causes c-Met induction. Oncotarget.

[30] Li M., Tang Y., Wu L., Mo F., Wang X., Li H., Qi R., Zhang H., Srivastava A., Ling C. (2017). The hepatocyte-specific HNF4alpha/miR-122 pathway contributes to iron overload-mediated hepatic inflammation. Blood.

[31] Hsu S.H., Wang B., Kota J., Yu J., Costinean S., Kutay H., Yu L., Bai S., La Perle K., Chivukula R.R. (2012). Essential metabolic, anti-inflammatory, and anti-tumorigenic functions of miR-122 in liver. J. Clin. Investig..

[32] Tsai W.C., Hsu S.D., Hsu C.S., Lai T.C., Chen S.J., Shen R., Huang Y., Chen H.C., Lee C.H., Tsai T.F. (2012). MicroRNA-122 plays a critical role in liver homeostasis and hepatocarcinogenesis. J. Clin. Investig..

[33] Takaki Y., Saito Y., Takasugi A., Toshimitsu K., Yamada S., Muramatsu T., Kimura M., Sugiyama K., Suzuki H., Arai E. (2014). Silencing of microRNA-122 is an early event during hepatocarcinogenesis from non-alcoholic steatohepatitis. Cancer Sci..

[34] Shim S.H., Xia C., Zhong G., Babcock H.P., Vaughan J.C., Huang B., Wang X., Xu C., Bi G.Q., Zhuang X. (2012). Super-resolution fluorescence imaging of organelles in live cells with photoswitchable membrane probes. Proc. Natl. Acad. Sci. USA.

[35] Burban A., Sharanek A., Guguen-Guillouzo C., Guillouzo A. (2018). Endoplasmic reticulum stress precedes oxidative stress in antibiotic-induced cholestasis and cytotoxicity in human hepatocytes. Free Radic. Biol. Med..

[36] Beriault D.R., Werstuck G.H. (2013). Detection and quantification of endoplasmic reticulum stress in living cells using the fluorescent compound, Thioflavin T. Biochim. Biophys. Acta.

[37] Xing Y., Higuchi K. (2002). Amyloid fibril proteins. Mech. Ageing Dev..

[38] Tardif K.D., Waris G., Siddiqui A. (2005). Hepatitis C virus, ER stress, and oxidative stress. Trends Microbiol..

[39] Waris G., Turkson J., Hassanein T., Siddiqui A. (2005). Hepatitis C virus (HCV) constitutively activates STAT-3 via oxidative stress: Role of STAT-3 in HCV replication. J. Virol..

[40] Gong G., Waris G., Tanveer R., Siddiqui A. (2001). Human hepatitis C virus NS5A protein alters intracellular calcium levels, induces oxidative stress, and activates STAT-3 and NF-kappa B. Proc. Natl. Acad. Sci. USA.

[41] Joyce M.A., Walters K.A., Lamb S.E., Yeh M.M., Zhu L.F., Kneteman N., Doyle J.S., Katze M.G., Tyrrell D.L. (2009). HCV induces oxidative and ER stress, and sensitizes infected cells to apoptosis in SCID/Alb-uPA mice. PLoS Pathog..

[42] Burdette D., Olivarez M., Waris G. (2010). Activation of transcription factor Nrf2 by hepatitis C virus induces the cell-survival pathway. J. Gen. Virol..

[43] Xu D., Xu M., Jeong S., Qian Y., Wu H., Xia Q., Kong X. (2018). The Role of Nrf2 in Liver Disease: Novel Molecular Mechanisms and Therapeutic Approaches. Front. Pharmacol..

[44] Ma Q. (2013). Role of nrf2 in oxidative stress and toxicity. Annu. Rev. Pharmacol. Toxicol..

[45] Nguyen T., Nioi P., Pickett C.B. (2009). The Nrf2-antioxidant response element signaling pathway and its activation by oxidative stress. J. Biol. Chem..

[46] Mitsuishi Y., Motohashi H., Yamamoto M. (2012). The Keap1-Nrf2 system in cancers: Stress response and anabolic metabolism. Front. Oncol..

[47] Kansanen E., Kuosmanen S.M., Leinonen H., Levonen A.L. (2013). The Keap1-Nrf2 pathway: Mechanisms of activation and dysregulation in cancer. Redox Biol..

[48] Vallianou I., Dafou D., Vassilaki N., Mavromara P., Hadzopoulou-Cladaras M. (2016). Hepatitis C virus suppresses Hepatocyte Nuclear Factor 4 alpha, a key regulator of hepatocellular carcinoma. Int. J. Biochem. Cell Biol..

[49] Li Z.Y., Xi Y., Zhu W.N., Zeng C., Zhang Z.Q., Guo Z.C., Hao D.L., Liu G., Feng L., Chen H.Z. (2011). Positive regulation of hepatic miR-122 expression by HNF4alpha. J. Hepatol..

[50] Zeng C., Wang R., Li D., Lin X.J., Wei Q.K., Yuan Y., Wang Q., Chen W., Zhuang S.M. (2010). A novel GSK-3 beta-C/EBP alpha-miR-122-insulin-like growth factor 1 receptor regulatory circuitry in human hepatocellular carcinoma. Hepatology.

[51] Kanda T., Goto T., Hirotsu Y., Moriyama M., Omata M. (2019). Molecular Mechanisms Driving Progression of Liver Cirrhosis towards Hepatocellular Carcinoma in Chronic Hepatitis B and C Infections: A Review. Int. J. Mol. Sci..

[52] Desai A., Sandhu S., Lai J.P., Sandhu D.S. (2019). Hepatocellular carcinoma in non-cirrhotic liver: A comprehensive review. World J. Hepatol..

[53] Stine J.G., Wentworth B.J., Zimmet A., Rinella M.E., Loomba R., Caldwell S.H., Argo C.K. (2018). Systematic review with meta-analysis: Risk of hepatocellular carcinoma in non-alcoholic steatohepatitis without cirrhosis compared to other liver diseases. Aliment. Pharmacol. Ther..

[54] Chandra P.K., Gunduz F., Hazari S., Kurt R., Panigrahi R., Poat B., Bruce D., Cohen A.J., Bohorquez H.E., Carmody I. (2014). Impaired expression of type I and type II interferon receptors in HCV-associated chronic liver disease and liver cirrhosis. PLoS ONE.

[55] Levy D.E., Darnell J.E. (2002). Stats: Transcriptional control and biological impact. Nat. Rev. Mol. Cell Biol..

[56] Calvisi D.F., Ladu S., Gorden A., Farina M., Conner E.A., Lee J.S., Factor V.M., Thorgeirsson S.S. (2006). Ubiquitous activation of Ras and Jak/Stat pathways in human HCC. Gastroenterology.

[57] Shen S., Niso-Santano M., Adjemian S., Takehara T., Malik S.A., Minoux H., Souquere S., Marino G., Lachkar S., Senovilla L. (2012). Cytoplasmic STAT3 represses autophagy by inhibiting PKR activity. Mol. Cell.

[58] Tacke R.S., Tosello-Trampont A., Nguyen V., Mullins D.W., Hahn Y.S. (2011). Extracellular hepatitis C virus core protein activates STAT3 in human monocytes/macrophages/dendritic cells via an IL-6 autocrine pathway. J. Biol. Chem..

[59] Kyrmizi I., Hatzis P., Katrakili N., Tronche F., Gonzalez F.J., Talianidis I. (2006). Plasticity and expanding complexity of the hepatic transcription factor network during liver development. Genes Dev..

[60] Odom D.T., Zizlsperger N., Gordon D.B., Bell G.W., Rinaldi N.J., Murray H.L., Volkert T.L., Schreiber J., Rolfe P.A., Gifford D.K. (2004). Control of pancreas and liver gene expression by HNF transcription factors. Science.

[61] Guzman-Lepe J., Cervantes-Alvarez E., Collin de l’Hortet A., Wang Y., Mars W.M., Oda Y., Bekki Y., Shimokawa M., Wang H., Yoshizumi T. (2018). Liver-enriched transcription factor expression relates to chronic hepatic failure in humans. Hepatol. Commun..

[62] Liu L., Yannam G.R., Nishikawa T., Yamamoto T., Basma H., Ito R., Nagaya M., Dutta-Moscato J., Stolz D.B., Duan F. (2012). The microenvironment in hepatocyte regeneration and function in rats with advanced cirrhosis. Hepatology.

[63] Safdar H., Cheung K.L., Vos H.L., Gonzalez F.J., Reitsma P.H., Inoue Y., van Vlijmen B.J. (2012). Modulation of mouse coagulation gene transcription following acute in vivo delivery of synthetic small interfering RNAs targeting HNF4alpha and C/EBPalpha. PLoS ONE.

[64] Zheng X.W., Kudaravalli R., Russell T.T., DiMichele D.M., Gibb C., Russell J.E., Margaritis P., Pollak E.S. (2011). Mutation in the factor VII hepatocyte nuclear factor 4alpha-binding site contributes to factor VII deficiency. Blood Coagul. Fibrinolysis.

[65] Inoue Y., Peters L.L., Yim S.H., Inoue J., Gonzalez F.J. (2006). Role of hepatocyte nuclear factor 4alpha in control of blood coagulation factor gene expression. J. Mol. Med..

[66] Bandiera S., Pfeffer S., Baumert T.F., Zeisel M.B. (2015). miR-122-a key factor and therapeutic target in liver disease. J. Hepatol..

[67] Trebicka J., Anadol E., Elfimova N., Strack I., Roggendorf M., Viazov S., Wedemeyer I., Drebber U., Rockstroh J., Sauerbruch T. (2013). Hepatic and serum levels of miR-122 after chronic HCV-induced fibrosis. J. Hepatol..

[68] Cermelli S., Ruggieri A., Marrero J.A., Ioannou G.N., Beretta L. (2011). Circulating microRNAs in patients with chronic hepatitis C and non-alcoholic fatty liver disease. PLoS ONE.

[69] Sarasin-Filipowicz M., Krol J., Markiewicz I., Heim M.H., Filipowicz W. (2009). Decreased levels of microRNA miR-122 in individuals with hepatitis C responding poorly to interferon therapy. Nat. Med..

[70] Hamdane N., Juhling F., Crouchet E., El Saghire H., Thumann C., Oudot M.A., Bandiera S., Saviano A., Ponsolles C., Roca Suarez A.A. (2019). HCV-Induced Epigenetic Changes Associated with Liver Cancer Risk Persist After Sustained Virologic Response. Gastroenterology.

[71] Perez S., Kaspi A., Domovitz T., Davidovich A., Lavi-Itzkovitz A., Meirson T., Alison Holmes J., Dai C.Y., Huang C.F., Chung R.T. (2019). Hepatitis C virus leaves an epigenetic signature post cure of infection by direct-acting antivirals. PLoS Genet..

[72] El-Araby R.E., Khalifa M.A., Zoheiry M.M., Zahran M.Y., Rady M.I., Ibrahim R.A., El-Talkawy M.D., Essawy F.M. (2019). The interaction between microRNA-152 and DNA methyltransferase-1 as an epigenetic prognostic biomarker in HCV-induced liver cirrhosis and HCC patients. Cancer Gene Ther..

[73] Chandra P.K., Bao L., Song K., Aboulnasr F.M., Baker D.P., Shores N., Wimley W.C., Liu S., Hagedorn C.H., Fuchs S.Y. (2014). HCV infection selectively impairs type I but not type III IFN signaling. Am. J. Pathol..

[74] Florczyk U., Czauderna S., Stachurska A., Tertil M., Nowak W., Kozakowska M., Poellinger L., Jozkowicz A., Loboda A., Dulak J. (2011). Opposite effects of HIF-1alpha and HIF-2alpha on the regulation of IL-8 expression in endothelial cells. Free Radic. Biol. Med..

[75] Son Y.O., Pratheeshkumar P., Roy R.V., Hitron J.A., Wang L., Zhang Z., Shi X. (2014). Nrf2/p62 signaling in apoptosis resistance and its role in cadmium-induced carcinogenesis. J. Biol. Chem..

[76] Xu H., Xu S.J., Xie S.J., Zhang Y., Yang J.H., Zhang W.Q., Zheng M.N., Zhou H., Qu L.H. (2019). MicroRNA-122 supports robust innate immunity in hepatocytes by targeting the RTKs/STAT3 signaling pathway. Elife.

[77] Van Renne N., Suarez A.A.R., Duong F.H.T., Gondeau C., Calabrese D., Fontaine N., Ababsa A., Bandiera S., Croonenborghs T., Pochet N. (2018). miR-135a-5p-mediated downregulation of protein tyrosine phosphatase receptor delta is a candidate driver of HCV-associated hepatocarcinogenesis. Gut.

[78] Chen Y.N., Shen A., Rider P.J., Yu Y., Wu K.L., Mu Y.X., Hao Q., Liu Y.L., Gong H., Zhu Y. (2011). A liver-specific microRNA binds to a highly conserved RNA sequence of hepatitis B virus and negatively regulates viral gene expression and replication. FASEB J..

[79] Luna J.M., Scheel T.K.H., Danino T., Shaw K.S., Mele A., Fak J.J., Nishiuchi E., Takacs C.N., Catanese M.T., de Jong Y.P. (2015). Hepatitis C Virus RNA Functionally Sequesters miR-122. Cell.

[80] Hyrina A., Olmstead A.D., Steven P., Krajden M., Tam E., Jean F. (2017). Treatment-Induced Viral Cure of Hepatitis C Virus-Infected Patients Involves a Dynamic Interplay among three Important Molecular Players in Lipid Homeostasis: Circulating microRNA (miR)-24, miR-223, and Proprotein Convertase Subtilisin/Kexin Type 9. Ebiomedicine.

[81] Jopling C.L., Yi M.K., Lancaster A.M., Lemon S.M., Sarnow P. (2005). Modulation of hepatitis C virus RNA abundance by a liver-specific microRNA. Science.

[82] Mylroie H., Dumont O., Bauer A., Thornton C.C., Mackey J., Calay D., Hamdulay S.S., Choo J.R., Boyle J.J., Samarel A.M. (2015). PKC epsilon-CREB-Nrf2 signalling induces HO-1 in the vascular endothelium and enhances resistance to inflammation and apoptosis. Cardiovasc. Res..

[83] Gao H.W., Guo R.F., Speyer C.L., Reuben J., Neff T.A., Hoesel L.M., Riedemann N.C., McClintock S.A., Sarma J.V., Van Rooijen N. (2004). Stat3 activation in acute lung injury. J. Immunol..

[84] Tawani A., Amanullah A., Mishra A., Kumar A. (2016). Evidences for Piperine inhibiting cancer by targeting human G-quadruplex DNA sequences. Sci. Rep..

[85] Mitchell P.S., Parkin R.K., Kroh E.M., Fritz B.R., Wyman S.K., Pogosova-Agadjanyan E.L., Peterson A., Noteboom J., O’Briant K.C., Allen A. (2008). Circulating microRNAs as stable blood-based markers for cancer detection. Proc. Natl. Acad. Sci. USA.

