# Supplementary material for: Hepatic Stress Response in HCV Infection Promotes STAT3-Mediated Inhibition of HNF4A-miR-122 Feedback Loop in Liver Fibrosis and Cancer Progression
Source: Cancers (Basel). 2019 Sep 20;11(10):1407. doi: 10.3390/cancers11101407 (PMC6827087; doi:10.3390/cancers11101407)
Supplement: Supplementary file 1 [file cancers-11-01407-s001.pdf]

**Supplementary Materials**

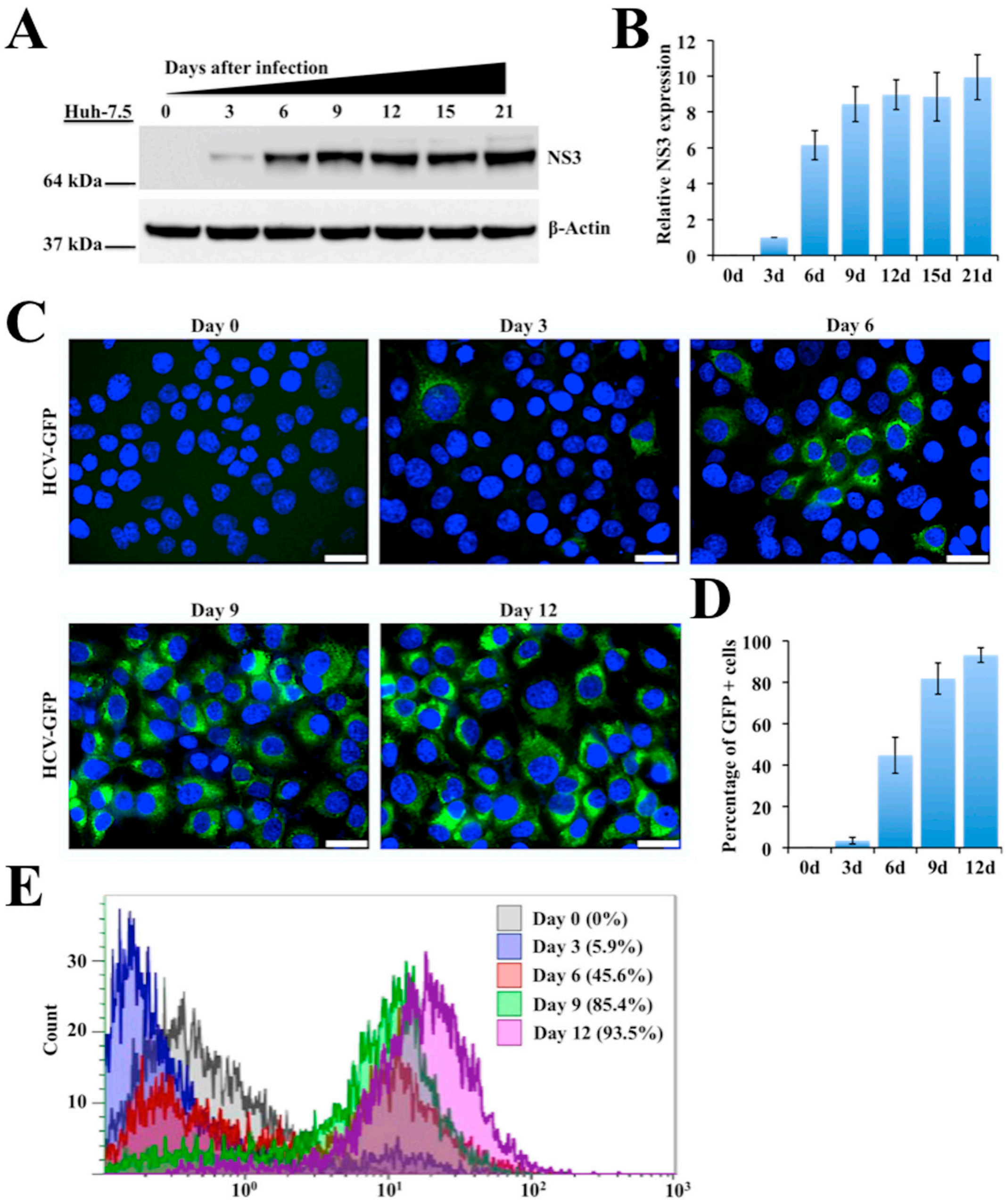


**Figure S1.** Persistent HCV replication model in permissive Huh-7.5 liver cells using HCV-GFP chimera virus. Huh-7.5 liver cells in culture were infected with HCV-GFP virus (JFH-AM120) at a MOI of 0.1 by overnight incubation. The next day cells were incubated with fresh media with 10% FBS. Infected culture was maintained for more than 21 day with regular medium change at a three-days interval. (**A**). Shown is the expression of NS3 protein in Huh-7.5 cells infected with HCV-GFP virus. (**B**). Shown are the Western blot quantification data by ImageJ software. Data of Western blot results repeated in three separate experiments. (**C**). Shown is the expression of NS5A-GFP chimera protein with DAPI staining in Huh-7.5 cells infected with HCV-GFP virus over 12 days. (**D**). Quantification of NS5A-GFP expression by ImageJ

software. (**E**). Flow cytometry analysis shows expression of GFP positive cells in infected culture at day 0, 3, 6, 9, and 12 days. Original magnification, ×60. Scale bars = 20 μm. Error bars represent the standard deviation (SD) of three experiments.

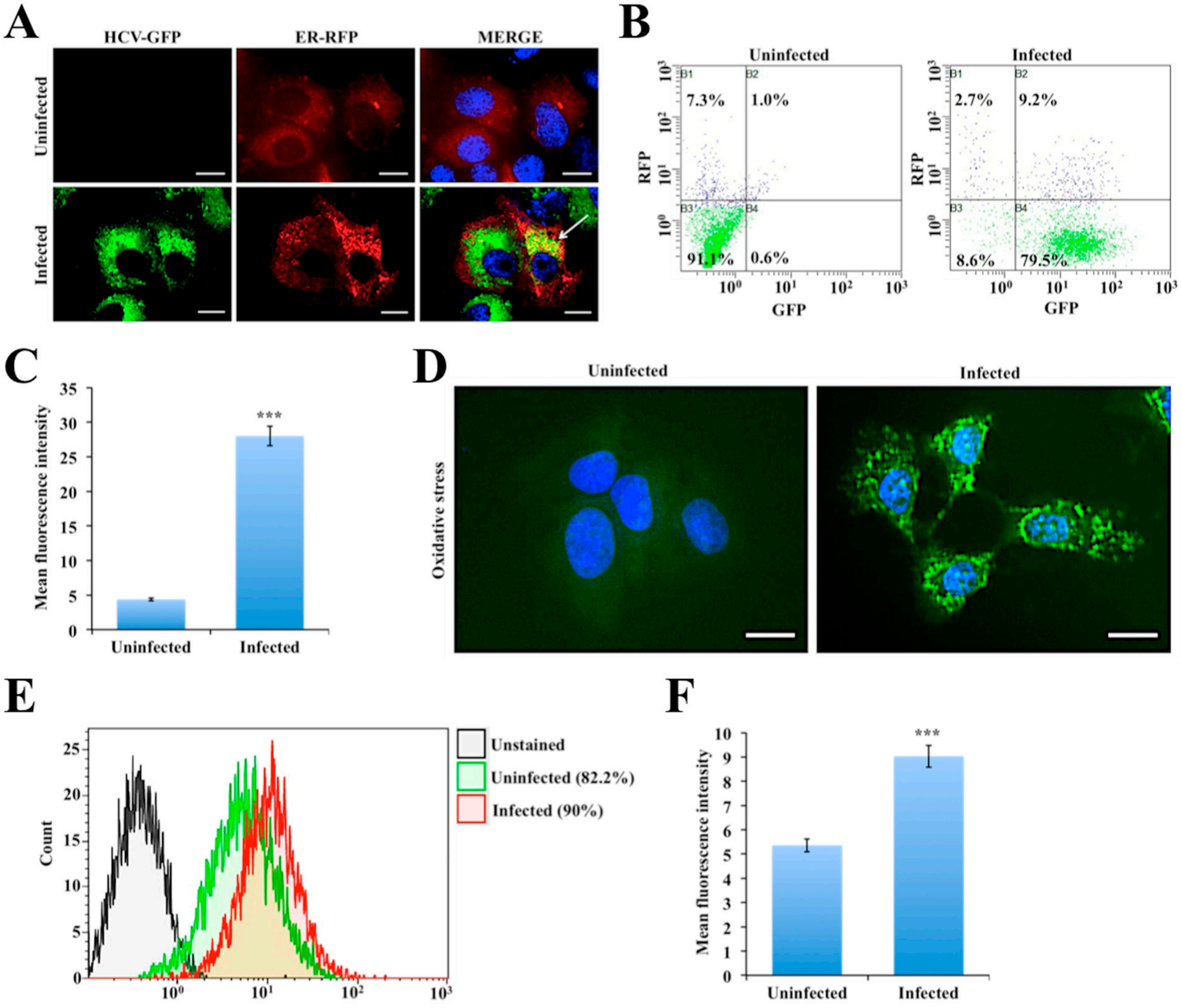


**Figure S2.** Liver cell imaging showing co-localization of HCV-GFP and Red-fluorescence protein fusion with KDEL-ER retention signal in the HCV-infected Huh-7.5 cells day 9. Infected and uninfected Huh-7.5 cells on day 9 were transfected with CellLight ER-RFP, BacMam 2.0 fusion construct of ER-signal overnight. This fusion construct carries sequence of calreticulin and KDEL (ER retention signal) and tags RFP that provide accurate targeting the RFP expression in the ER. (**A**). Shown is the fluorescence imaging after 24 hours showing the staining intensity between uninfected and infected Huh-7.5 culture. The HCV-GFP fluorescence colocalize with ER-RFP, suggesting that HCV replication in the ER. The arrow indicates co-localization. (**B**). The amplification of co-localization signal of HCV-GFP with RFP fluorescence. Most of the RFP fluorescence is colocalized with GFP. (**C**). Shown are the data of three separate analyses showing increased co-localization of RFP-GFP in HCV culture. (**D**). Oxidative stress fluorescence in uninfected Huh-7.5 liver and HCV infected cells on day 9. Oxidative stress due to generation of ROS was measured using conventional fluorescence microscopy. In this assay, the nonfluorescent dye (H2DCFDA) is oxidized to a fluorescence green (DFA) by ROS only in HCV infected cells. (**E**). Shown is the flow cytometric analysis of oxidized fluorescence of uninfected and infected Huh-7.5 cells. (**F**). Quantification of

fluorescent intensity in three different areas of uninfected and infected culture by ImageJ software. Fluorescence values are proportional to intracellular ROS. Original magnification, ×60. Scale bars = 40 μm. The results are expressed as the mean ± standard deviation (SD) of three experiments. Error bars represent SD. P values were calculated by ANOVA between different groups. *** $p$-value < 0.001.

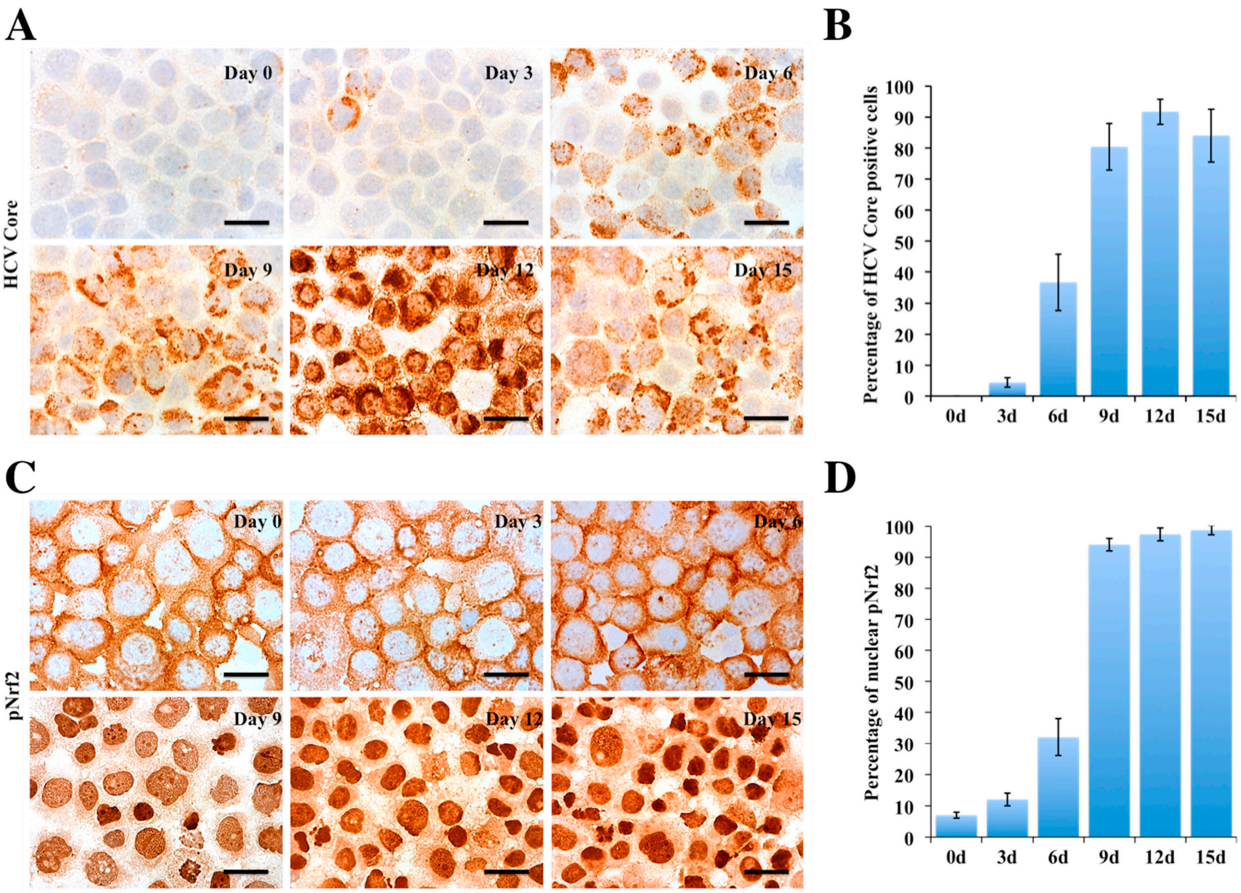


**Figure S3.** Persistent replication of HCV-GFP chimera induces NRF2 activation and nuclear translocation. (**A**). Shown is the immunocytochemical staining of pNRF2 in HCV-infected Huh-7.5 cells at different days after HCV infection. (**B**). Quantification of nuclear NRF2 positive infected Huh-7.5 cells using ImageJ software. Three high-power fields were measured. (**C**). Shown is the Immunocytochemical staining of core protein in HCV-infected Huh-7.5 cells at different days. (**D**). Quantification of brown positive cytoplasmic core staining in infected Huh-7.5 cells using ImageJ software. Error bars represent the standard deviation (SD) of three experiments. Three high-power fields were measured. Original magnification, ×100. Scale bars = 200 μm.

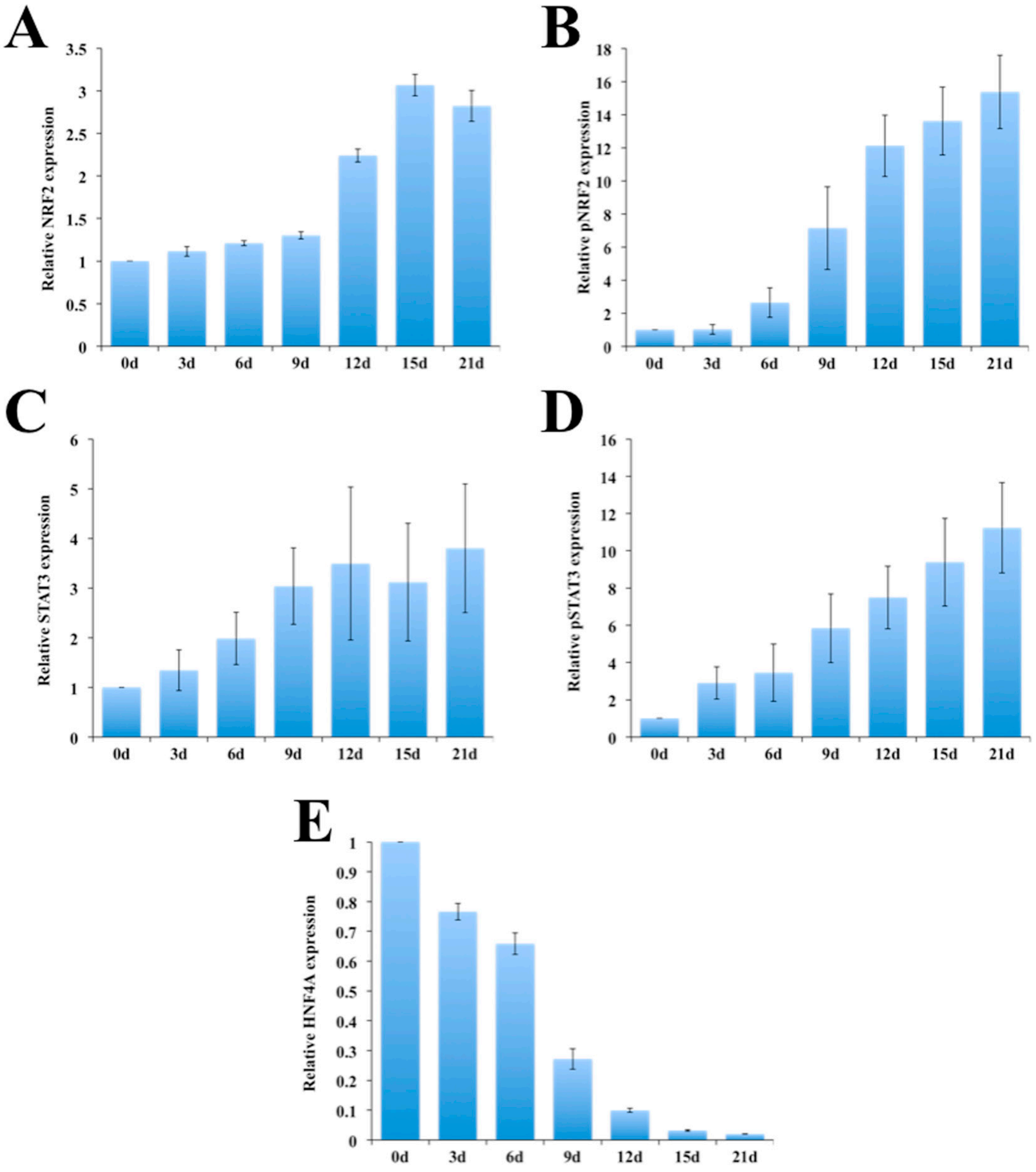


**Figure S4.** (**A**–**E**) Western blot band intensity of Figure 1 was quantified by ImageJ software. First, band intensity of individual protein and loading controls were scanned. The ratio of net band values over the net loading control was calculated. The results were compared through bar graph. Error bars represent the standard deviation (SD) of three experiments.

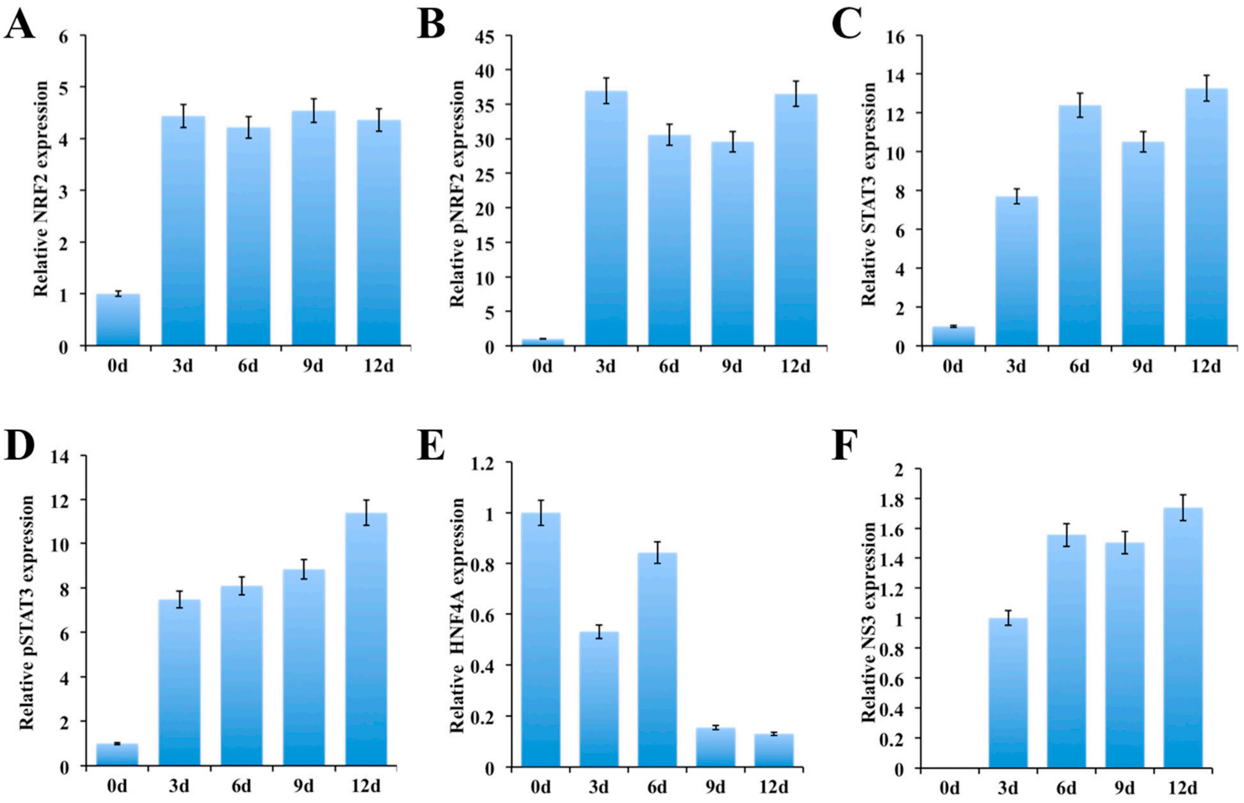


**Figure S5.** (**A**–**F**) Western blot band intensity of Figure 2A were quantified using ImageJ software. The results were compared using bar graph. Error bars represent the standard deviation (SD) of three experiments.

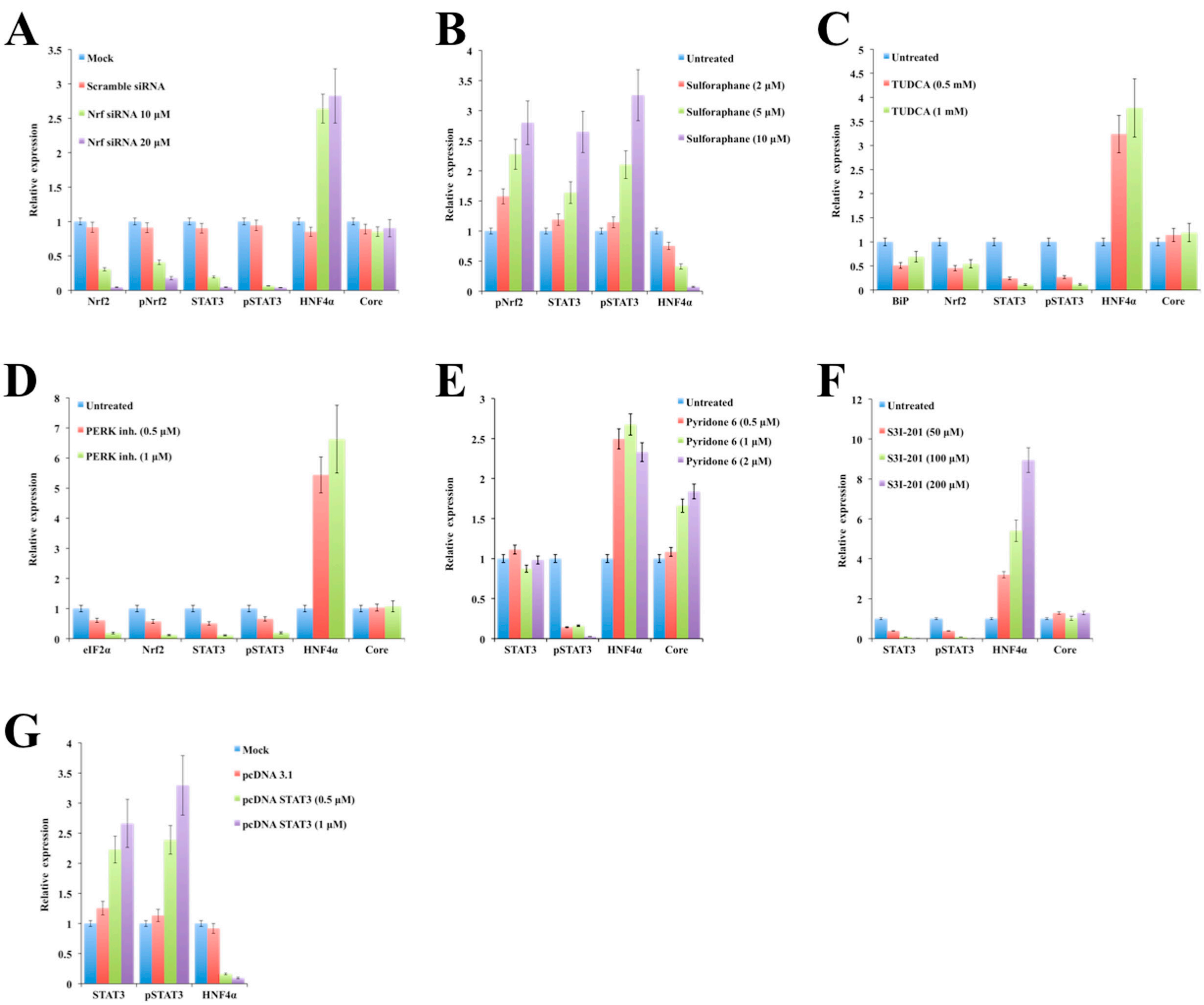


**Figure S6.** (**A**–**G**) Western blot band intensity of Figure 3 was quantified using ImageJ software and results were compared using bar graph. Error bars represent the standard deviation (SD) of three experiments.

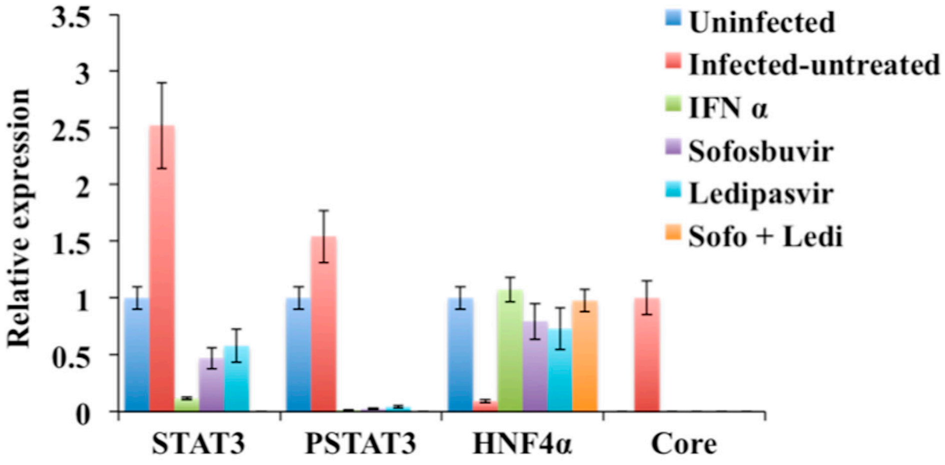


**Figure S7.** Western blot band intensity of Figure 4E were quantified using ImageJ software and results were compared using bar graph. Error bars represent the standard deviation (SD) of three experiments.
